# Visualization of aging-associated chromatin alterations with an engineered TALE system

**DOI:** 10.1038/cr.2017.18

**Published:** 2017-01-31

**Authors:** Ruotong Ren, Liping Deng, Yanhong Xue, Keiichiro Suzuki, Weiqi Zhang, Yang Yu, Jun Wu, Liang Sun, Xiaojun Gong, Huiqin Luan, Fan Yang, Zhenyu Ju, Xiaoqing Ren, Si Wang, Hong Tang, Lingling Geng, Weizhou Zhang, Jian Li, Jie Qiao, Tao Xu, Jing Qu, Guang-Hui Liu

**Affiliations:** 1 National Laboratory of Biomacromolecules, CAS Center for Excellence in Biomacromolecules, Institute of Biophysics, Chinese Academy of Sciences, Beijing 100101, China; 2 University of Chinese Academy of Sciences, Beijing 100049, China; 3 State Key Laboratory of Stem Cell and Reproductive Biology, Institute of Zoology, Chinese Academy of Sciences, Beijing 100101, China; 4 Gene Expression Laboratory, Salk Institute for Biological Studies, 10010 North Torrey Pines Road, La Jolla, CA 92037, USA; 5 Department of Gynecology and Obstetrics, Peking University Third Hospital, Beijing 100191, China; 6 The MOH Key Laboratory of Geriatrics, Beijing Hospital, National Center of Gerontology, Beijing 100730, China; 7 Department of Pediatrics, Beijing Shijitan Hospital Capital Medical University, Peking University Ninth School of Clinical Medicine, Beijing 100038, China; 8 Key Laboratory of Regenerative Medicine of Ministry of Education, Institute of Aging and Regenerative Medicine, Jinan University, Guangzhou, Guangdong 510632, China; 9 Institute of Aging Research, Hangzhou Normal University School of Medicine, Hangzhou, Zhejiang 311121, China; 10 Department of Pathology, Carver College of Medicine, University of Iowa, Iowa City, IA 52242, USA; 11 Beijing Institute for Brain Disorders, Beijing 100069, China

**Keywords:** thioredoxin-fused TALE, aging, ribosomal DNA repeat, telomere, centromere

## Abstract

Visualization of specific genomic loci in live cells is a prerequisite for the investigation of dynamic changes in chromatin architecture during diverse biological processes, such as cellular aging. However, current precision genomic imaging methods are hampered by the lack of fluorescent probes with high specificity and signal-to-noise contrast. We find that conventional transcription activator-like effectors (TALEs) tend to form protein aggregates, thereby compromising their performance in imaging applications. Through screening, we found that fusing thioredoxin with TALEs prevented aggregate formation, unlocking the full power of TALE-based genomic imaging. Using thioredoxin-fused TALEs (TTALEs), we achieved high-quality imaging at various genomic loci and observed aging-associated (epi) genomic alterations at telomeres and centromeres in human and mouse premature aging models. Importantly, we identified attrition of ribosomal DNA repeats as a molecular marker for human aging. Our study establishes a simple and robust imaging method for precisely monitoring chromatin dynamics *in vitro* and *in vivo*.

## Introduction

In the human nuclear genome, ∼3.2 billion base pairs of DNA are tightly packed into 23 chromosome pairs of varying size. Although we can obtain sequence information for any genomic locus with ease, we are still in the early stages of unraveling the organization of the human genome and the spatiotemporal relationships between genomic loci in three-dimensional (3D), which will consequently improve our understanding of genetic and epigenetic regulation during cell differentiation, aging, and pathophysiological processes^1,2,3,4,5,6,7^. The importance of maintaining a proper 3D genome topology is underscored by recent discoveries that aging and several severe human diseases can be attributed to genomic disorganization^7,8,9^. Therefore, exploring the spatial organization of human chromatin and its dynamic interactions with protein and RNA regulators emerges as an important part of understanding the cellular processes underlying aging and human diseases^3,10,11^.

Despite recent advances, there is a lack of imaging tools suitable for accurate and long-term tracking of topological changes at specific genomic loci. A series of tools have been reported for visualization of repetitive DNA sequences or specific gene loci in fixed or live human cells, but they suffer from several limitations. (1) Fluorescent Lac or Tet operator and repressor systems have been used to visualize target genomic sequences in live cells^12,13^, but the integration of large artificial sequences (∼10 kb) into the genomic region of interest suffers from low efficiency and a risk of disturbing genomic structures. (2) Fluorescent *in situ* hybridization (FISH) has been widely used to study nuclear localization of specific sequences and genomic aberrations^14^, but can only be performed on fixed cells after DNA denaturation. (3) Recently, the CRISPR/dCas9 system has been adapted for visualization of specific genomic loci (e.g., protein-coding mucin genes such as *MUC4*) and repetitive telomere sequences in live cells^15,16,17^. However, low signal-to-noise ratios, inefficient delivery of the bulky Cas9 and single-guide RNA (sgRNA) constructs, and potential cellular stresses caused by overloading sgRNA have limited their utility^18,19^. (4) Fluorescent protein-labeled transcription activator-like effectors (TALEs) have been used for live imaging of genomic repetitive sequences^17,20,21^. TALEs are proteins secreted by *Xanthomonas* bacteria that infect various plant species. TALEs are DNA-binding proteins that contain tandem 33- to 35-amino acid (aa) repeats, each of which specifically recognizes and binds to a single target DNA base^22,23^. The smaller size of TALEs and simple correlation between TALEs and target DNA bases makes them extremely useful for designing artificial constructs capable of recognizing genomic sequences in diverse experimental systems. Indeed, engineered TALEs have been harnessed for a variety of applications, including genome editing (when fused to the cleavage domain of FokI nuclease or to meganucleases)^24^ and design of customized transcriptional modulators^25,26^ and recombinases^27^. Due to their relatively small size, fluorescently tagged TALEs have been used as small protein probes to track specific genomic DNA sequences, especially within telomeres and centromeres, in live cells^20,21,28,29^. Despite these advances, a careful validation of TALE-based imaging in different cellular systems is still needed. Importantly, TALE- and Cas9/sgRNA-based imaging systems have seldom been tested in physiological and pathological contexts such as human aging.

Here we report that conventional TALEs frequently form large aggregates in human cells, thereby compromising their imaging efficiency in various cell types examined. To overcome this barrier, we developed a novel thioredoxin-fused TALE (TTALE) imaging system that can effectively eliminate aggregates and enable high-contrast visualization of the 3D dynamics of specific genomic structures under diverse physiological and pathological contexts (e.g., aging) across a wide range of cell types *in vitro* and *in vivo*.

## Results

### Precise visualization of telomeres and centromeres using TTALEs

**Figure 1:**
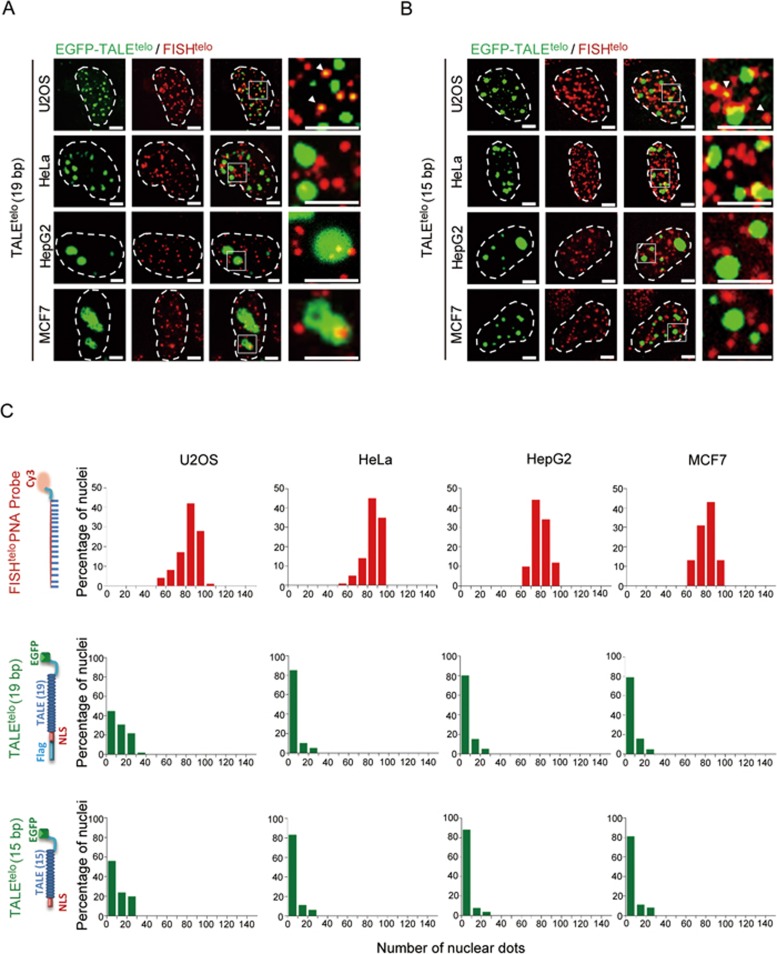
Conventional TALEs were insufficient to precisely track telomeres in four human tumor cell lines tested. **(A-B)** Co-localization analysis of telomeric FISH (red) and EGFP-TALE^telo^ (green) signals in the indicated human cell lines (U2OS, HeLa, HepG2, and MCF7). TALE^telo^ was designed using a 19-bp **(A)** or a 15-bp **(B)** telomeric repetitive sequence. Dashed lines indicate the nuclear boundaries; arrowheads indicate overlapping signals. Scale bars, 5 μm. **(C)** Histograms showing numbers of EGFP-TALE^telo^(15 bp)-, EGFP-TALE^telo^(19 bp)-, and telomeric FISH-positive nuclear dots in each cell line. *n* = 50 nuclei per cell line.

To adapt TALEs for visualizing specific genomic locus, we generated an EGFP-tagged TALE construct targeting a 19-bp telomeric DNA repeat (5′-TAACCCTAACCCTAACCCT-3′ referred to as TALE^telo^). This construct was transiently introduced into four human tumor cell lines (U2OS, HeLa, HepG2, and MCF7). To examine the efficiency of TALE-based fluorescence imaging of telomeric sequences, 3D-FISH with a telomeric peptide nucleic acid (PNA) probe was used to visualize the 3D distribution of telomeres inside the nucleus^7^. Approximately 20% of FISH-positive foci overlapped with EGFP-TALE^telo^ in U2OS cells. In other cell types examined, EGFP-TALE^telo^ appeared mostly as large bright foci (referred to hereafter as “aggregates”) distinct from telomeric foci, with only a small percentage (< 5%) of foci co-localized with the FISH signals (Figure 1A and 1C). In addition, an EGFP-tagged TALE against a 19-bp centromeric satellite DNA repeat (5′-TCCATTCCATTCCATTCCA-3′), referred to as TALE^centro^, also failed to faithfully identify centromeres in the same cell types, as determined by 3D-FISH with a centromeric PNA probe (Supplementary information, Figure S1A and S1B). Similar results were also observed using previously reported EGFP-TALEs against a 15-bp telomeric repeat (5′-TAACCCTAACCCTAA-3′ Figure 1B and 1C) or a 20-bp centromeric repeat (5′-TAGACAGAAGCATTCTCAGA-3′ data not shown)^20^. We further verified that these aggregates were not localized in nucleoli (Supplementary information, Figure S1C-S1D). These data indicate that conventional TALEs are inefficient for tracking human telomeric and centromeric DNAs.

**Figure 2:**
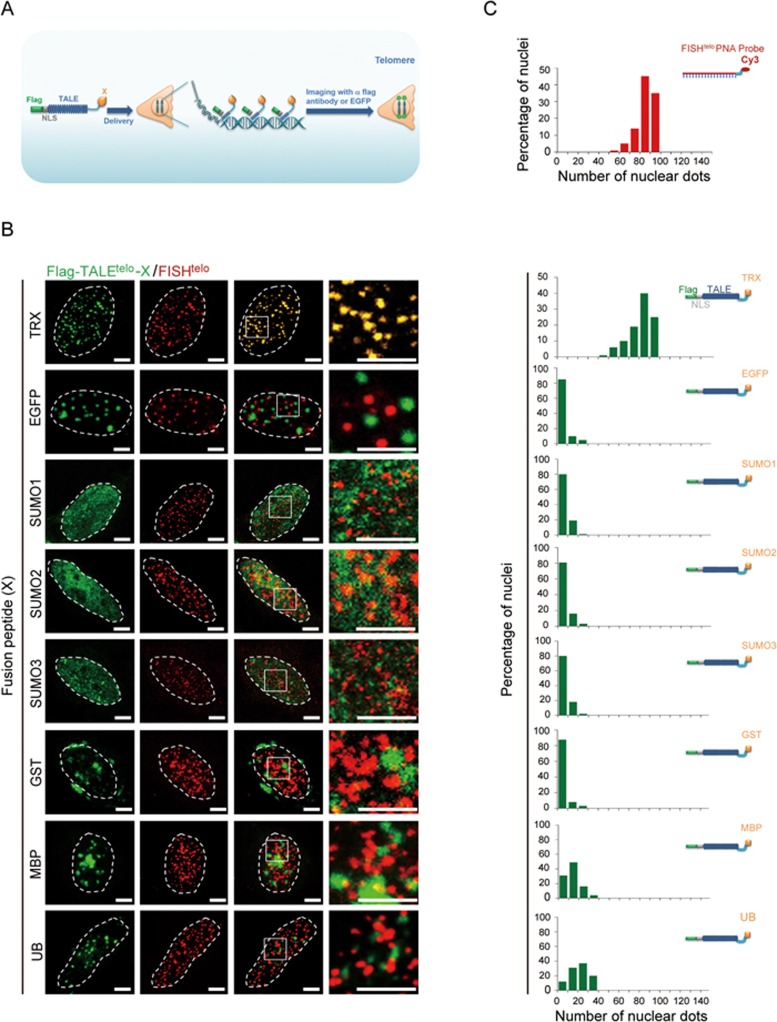
Precise labeling of telomeres with thioredoxin-fused TALEs (TTALEs). **(A)** Schematic illustration of TALEs fused with various solubility-enhancing peptides (X) to label telomeres. **(B)** Co-localization analysis of telomeric FISH (red) and Flag-TALE^telo^ (green) fused with the indicated peptides in HeLa cells. Engineered TALE^telo^ was visualized by immunostaining with anti-Flag antibody. Representative images using various fusion partners show that precise co-localization with telomeric FISH signals was obtained only using the thioredoxin-fused TALE^telo^ (TTALE^telo^). Dashed lines indicate the nuclear boundary. Scale bars, 5 μm. **(C)** Histograms showing numbers of telomeric FISH- and peptide-fused TALE^telo^(19 bp)-positive dots in nuclei of HeLa cells. *n* = 50 nuclei per group.

**Figure 3:**
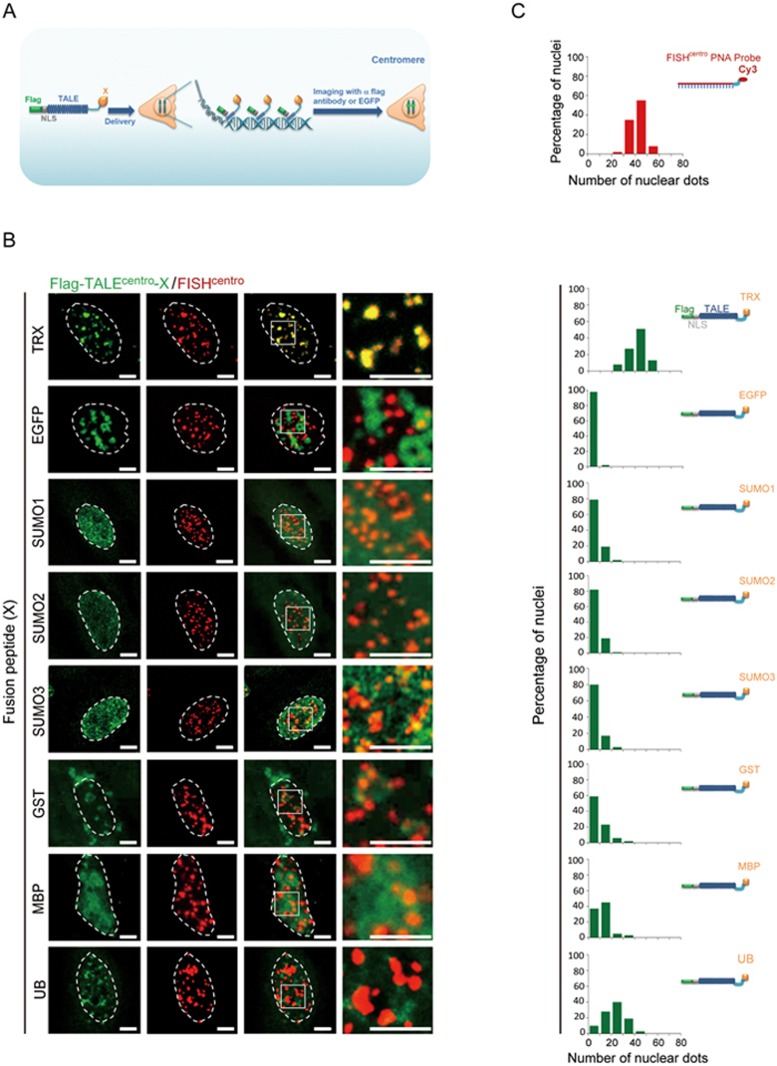
Precise labeling of centromeres with TTALEs. **(A)** Schematic illustration of TALEs fused with various solubility-enhancing peptides (X) to label centromeres. **(B)** Co-localization analysis of centromeric FISH (red) and Flag-TALE^centro^ (green) fused with the indicated peptides in HeLa cells. Engineered TALE^centro^ was visualized by immunostaining with anti-Flag antibody. Representative images show precise co-localization of centromeric FISH (red) and TRX-fused TALE^centro^ (TTALE^centro^) signals. Dashed lines indicate the nuclear boundary. Scale bars, 5 μm. **(C)** Histograms showing numbers of centromeric FISH- and peptide-fused TALE^centro^(19 bp)-positive dots in nuclei of HeLa cells. *n* = 50 nuclei per group.

TALEs are composed of multiple highly repetitive modules, a feature that likely predisposes them to self-assemble into bulky protein aggregates especially when being simultaneously tethered to multiple copies of genomic repetitive DNA sequences, preventing their binding to cognate DNA sequences. We thus screened a panel of peptides known to facilitate expression of insoluble proteins in *Escherichia coli*^30^, and fused them with TALEs (Figure 2A). We used the following fusion partners for initial screening: ubiquitin (UB), small ubiquitin-related modifier (SUMO), glutathione S-transferase (GST), maltose-binding protein (MBP), and thioredoxin (TRX) (Figure 2B and 2C). Among all peptides tested in telomeres, TRX was the best candidate, as the use of TRX-fused TALE^telo^ (TTALE^telo^) led to nearly perfect co-localization of telomeric FISH and TTALE^telo^ signals (Figure 2B and 2C). Likewise, the TRX-fused TALE^centro^ (TTALE^centro^) also yielded specific signals precisely marking centromeres and overlapping with centromeric FISH signals (Figure 3A-3C) as well as the centromeric protein CENPA (Supplementary information, Figure S2A).

TRX is a small oxidoreductase whose chaperone-like activity has been used to improve the cellular solubility of its fusion partners^30,31^. We thus investigated whether the redox-catalytic activity of TRX is involved in the precise recognition exhibited by TTALEs. We generated a redox-inactivated TRX mutant by replacing cysteines 32 and 35 with serines^32,33,34^. Although mutant TTALE^telo^ and TTALE^centro^ were expressed at similar levels to their wild-type (WT) counterparts (Supplementary information, Figure S2B), they failed to specifically label telomeric and centromeric loci, respectively (Supplementary information, Figure S2C), indicating that the redox activity of TRX is required for labeling genomic loci. In addition, we investigated whether the precise genomic labeling of TTALEs indirectly relies on the change of nuclear reductive status induced by overexpression of TRX. To this end, HeLa cells were co-transfected with unmodified TALE^telo^ or TALE^centro^ together with GAL4-TRX, a nuclear-localized TRX^33,35,36^ (Supplementary information, Figure S2D). Co-expression of conventional TALEs with non-fused TRX failed to target TALEs to their corresponding genomic loci (Supplementary information, Figure S2E-S2G). We treated the cells with *N*-acetyl-cysteine (NAC), a strong antioxidant that increases cellular reductive levels^37^, and found that suppression of oxidative stress by NAC did not result in correct genomic targeting by conventional TALEs (Supplementary information, Figure S2H). Finally, we compared the effect of TRX with another small redox protein, glutaredoxin (GRX)^38,39^. The GRX-fused TALE^telo^ did not exhibit telomere-specific distribution (Supplementary information, Figure S2I). Therefore, our results support a specific chaperone role of TRX when fused with TALE.

### Comparison of TTALE with the dCas9/sgRNA system for imaging specific genomic loci

Given that dCas9/sgRNA has also been used as a tool to visualize specific genomic loci^15^, we next compared the imaging quality of TTALE with that of dCas9/sgRNA in HeLa cells (Supplementary information, Figure S3A). EGFP-TTALE^telo^ exhibited clearer and sharper fluorescence signals at telomeric loci compared with dCas9/sgRNA, with significantly lower background in the nucleoplasm (Supplementary information, Figure S3B and S3C). After normalization for background noise, the specific fluorescence intensity of EGFP-TTALE^telo^ was 4.21-fold higher than that of dCas9/sgRNA (Supplementary information, Figure S3D). In addition, flow cytometry showed a much higher transfection efficiency using EGFP-TTALE^telo^ compared with EGFP-dCas9/sgRNA in multiple cell types tested (Supplementary information, Figure S3E and S3F).

### Imaging chromatin dynamics during mitosis with TTALEs

**Figure 4:**
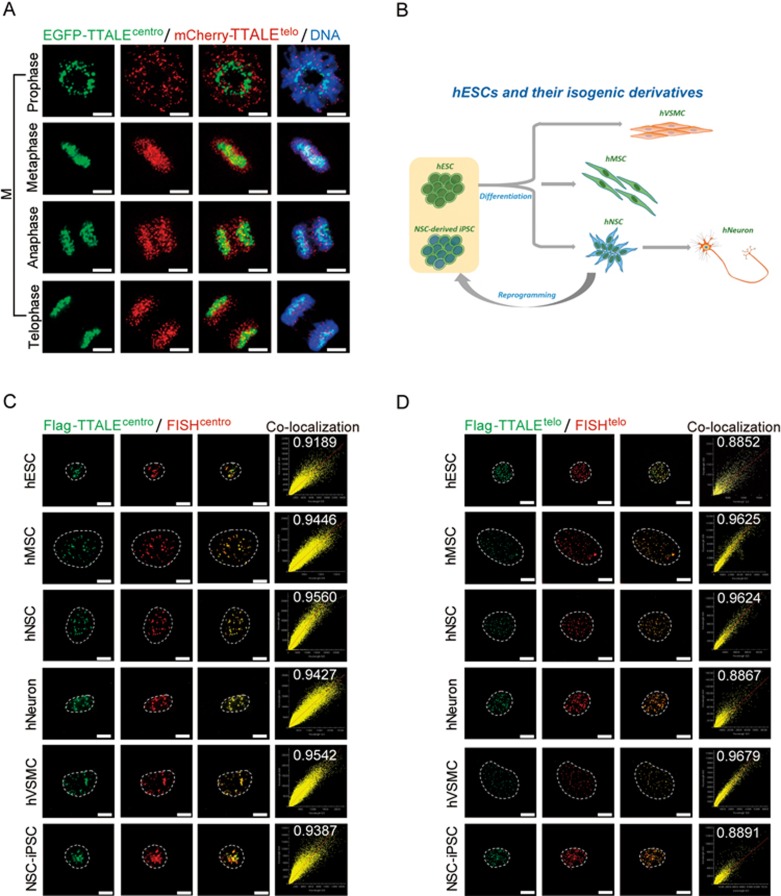
TTALE-mediated imaging of telomeres and centromeres in mitotic HeLa cells, hESCs, and the isogenic cell derivatives of hESCs. **(A)** Dynamic distribution of telomeres and centromeres at different stages of mitosis. Live HeLa cells co-expressing EGFP-TTALE^centro^ and mCherry-TTALE^telo^ were imaged at different mitotic phases. Scale bars, 5 μm. **(B)** Schematic diagram showing derivation of isogenic cell types from hESCs. hMSCs, hNSCs, and hVSMCs were differentiated from hESCs, and hNSCs were further differentiated into postmitotic neurons or reprogrammed into iPSCs. **(C-D)** Structured illumination microscopy (SIM) images showing co-localization of centromeric FISH (red) and Flag-TTALE^centro^ (green) **(C)** or telomeric FISH (red) and Flag-TTALE^telo^ (green) **(D)** signals in the indicated cell types. The number of each scatter plot represents the Pearson *r* value to show the linear correlation between FISH and TTALE^telo^ or TTALE^centro^ signals. Dashed lines indicate the nuclear boundary. Scale bars, 5 μm.

To visualize the dynamic distribution of telomeres and centromeres, we imaged HeLa cells at different mitotic phases using TTALE^telo^ and TTALE^centro^ probes. As in interphase cells, we observed nearly perfect overlap between TTALE^telo^ and telomeric FISH signals and between TTALE^centro^ and centromeric FISH signals during prophase, metaphase, anaphase, and telophase (Supplementary information, Figure S4A and S4B). In addition, telomeres and centromeres could be simultaneously visualized at all stages of mitosis by co-expression of mCherry-TTALE^telo^ and EGFP-TTALE^centro^(Figure 4A and Supplementary information, Movie S1). These data indicate that binding of TTALE^telo^ and TTALE^centro^ probes to telomeric and centromeric DNAs, respectively, is stable and specific throughout mitosis, and further suggest that the expression of TTALEs has minimal effect on mitosis in HeLa cells.

### TTALE-based imaging in human stem cells, differentiated cells, and oocytes

We differentiated human embryonic stem cells (ESCs) into two types of adult stem cells, neural stem cells (NSCs) and mesenchymal stem cells (MSCs), and two terminally differentiated cell types, vascular smooth muscle cells (VSMCs) and postmitotic neurons^7,8,37,40,41,42,43^ (Figure 4B and Supplementary information, Figure S5A-S5E). ESC-derived NSCs were also reprogrammed to generate induced pluripotent stem cells (iPSCs; Supplementary information, Figure S5F-S5G). All ESCs and their isogenic derivatives, the iPSCs, and five transformed human cell lines (HEK293, U2OS, HeLa, HepG2, and MCF7) were employed to evaluate imaging efficiency using EGFP-TTALEs. In all of these cell types, TTALE^telo^ and TTALE^centro^ signals perfectly co-localized with telomeric and centromeric FISH signals, respectively (Figure 4C and 4D, Supplementary information, Figure S4C and S4D), indicating that TTALEs are amenable to accurate and high-quality imaging for all human cells tested. Importantly, we also tested the performance of TTALE imaging using unfertilized human oocytes^44^. We microinjected EGFP-tagged TTALE^telo^ or TTALE^centro^ constructs directly into human oocytes and performed live cell imaging immediately after microinjection for 8-12 h. As in cultured cell lines, telomeric and centromeric signals were clearly visible (Supplementary information, Figure S4E, S4F and Movie S2).

### TTALE-based imaging of rDNA and single-gene loci

**Figure 5:**
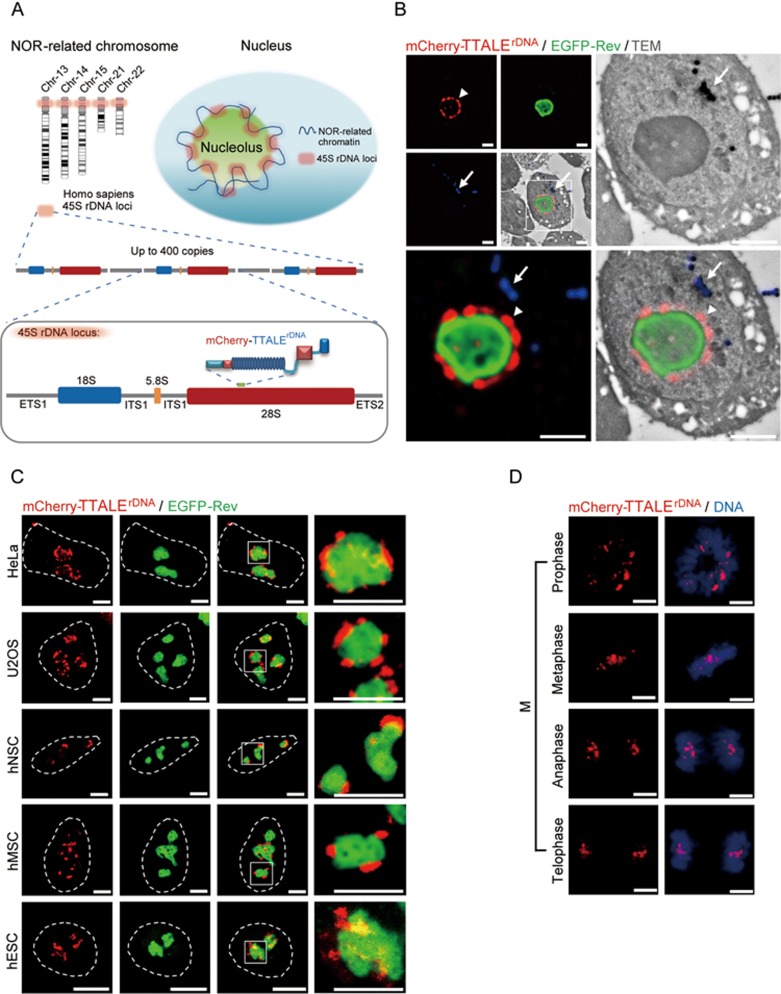
TTALE-based imaging of NOR-rDNAs. **(A)** Schematic diagram showing distribution and structural features of NOR-rDNAs in the human genome. **(B)** Co-localization analysis of mCherry-TTALE^rDNA^ (red) and EGFP-Rev (green, labeling nucleolus) signals captured by SIM-TEM. Arrowhead indicates mCherry-TTALE^rDNA^ signals at perinucleolar regions. Arrow indicates the fiducial marker (blue) for precise alignment of SIM and TEM images. Scale bars, 2 μm. **(C)** Live cell co-localization analysis of mCherry-TTALE^rDNA^ (red) and EGFP-Rev (green) in the indicated cell types. Dashed lines indicate the nuclear boundary. Scale bars, 5 μm. **(D)** Visualization of rDNA at different stages of mitosis in HeLa cells using mCherry-TTALE^rDNA^ (red). Hoechst was used to stain DNA (blue). Scale bars, 5 μm.

In the human genome, nucleolar organizer region (NOR)-related ribosomal DNAs (NOR-rDNAs) are composed of up to 400 repetitive DNA sequences^45^. Human 45S rDNA loci encoding 18S, 5.8S, and 28S rRNAs are organized as clusters within NORs. To visualize and monitor NOR-rDNAs, we generated a TTALE targeting a 19-bp DNA sequence (5′-TACCCTACTGATGATGTGT-3′) from 28S rDNA repeats (TTALE^rDNA^; Figure 5A). In MSCs, TTALE^rDNA^ exhibited a punctate staining pattern predominantly around nucleoli, whereas a conventional TALE targeting the same sequence labeled cytosolic aggregates (Supplementary information, Figure S6A and S6B). Signals from 3D-FISH probes completely overlapped with TTALE^rDNA^ signals at the endogenous 28S rDNA loci (Supplementary information, Figure S6D). Combining mCherry-TTALE^rDNA^, immunostaining with EGFP-Rev (a nucleolus-specific HIV protein^46^), nucleolin, and fibrillarin, and structured illumination microscopy-transmission electron microscopy (SIM-TEM) imaging with ultrahigh resolution, we further observed that the 28S rDNA loci were predominantly localized along the surface of the nucleolus or occasionally outside the nucleolus similar to pseudo-NORs^47,48^, and were rarely found inside the nucleolus (Figures 5B, Supplementary information, Figure S6C, S6D, and S6E). We further validated the TTALE^rDNA^-mediated high-quality imaging in diverse human cell lines (Figure 5C) and human oocytes (Supplementary information, Figure S6F), and at different mitotic phases in HeLa cells (Figure 5D). To simultaneously visualize telomeres, centromeres, and 28S rDNA sequences, hMSCs were co-transfected with mCherry-TTALE^telo^, YFP-TTALE^centro^, and CFP-TTALE^rDNA^. Our results demonstrate that the TTALE system is compatible with multiplex imaging of different genomic loci at the single-cell level (Supplementary information, Figure S6G). In addition to non-gene loci, we further demonstrated that TTALEs can be used for gene-coding genomic loci using *MUC4* as an example in interphase hMSCs and at different mitotic phases in HeLa cells (Supplementary information, Figure S6H-S6K)^15,16,49,50,51,52^.

### TTALE-based imaging to track genomic changes during human aging

**Figure 6:**
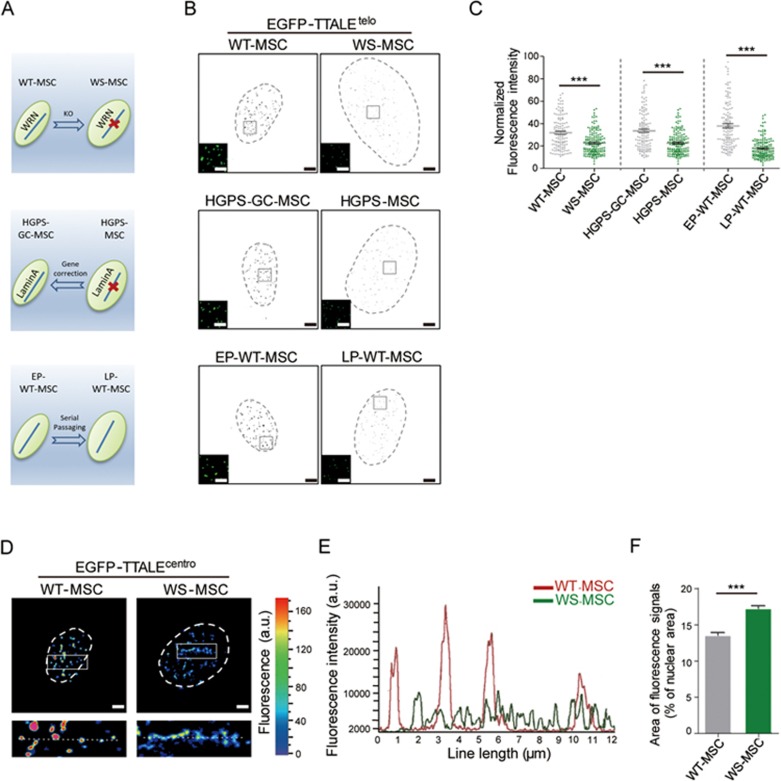
TTALE-mediated imaging of telomeres and centromeres during human stem cell aging. **(A)** Schematic diagram of three established human stem cell aging models. **(B)** SIM images showing EGFP-TTALE^telo^-labeled telomeres in WS-MSCs vs WT-MSCs at passage 6 (top), HGPS-MSCs vs HGPS-GC-MSCs at passage 8 (middle), and LP-WT-MSCs at passage 12 vs EP-WT-MSCs at passage 6 (bottom). Magnified images of the boxed regions are in the lower-left corner. Dashed lines indicate the nuclear boundary. Scale bars, 5 μm (wide-field images) or 10 μm (magnified images). **(C)** Decrease of EGFP-TTALE^telo^ fluorescence intensity with hMSC aging. The scatter plot displays the fluorescence intensity of EGFP-TTALE^telo^-labeled telomere puncta normalized by the average fluorescence intensity of the nucleus in the indicated cells. *n* = 50 nuclei per group; ^***^P < 0.001. **(D)** SIM images of centromeres labeled by EGFP-TTALE^centro^ in WS-MSCs and WT-MSCs. Bottom panels show magnified images of boxed regions from the top panels. Dashed lines indicate the nuclear boundary. Scale bars, 5 μm. **(E)** Intensity profiles of TTALE^centro^ signals across the dotted lines (12 μm in length) in the bottom panels of **D**. **(F)** Histogram showing the percentage of fluorescence-labeled area relative to nuclear area in the indicated cells. *n* = 50 nuclei per group; ^***^P < 0.001.

Human cellular aging is driven by both genomic and epigenomic alterations^3,7,8,37,40,42,43,53^. We next applied TTALE imaging to monitor genomic changes during human cellular aging. Telomere attrition is an established hallmark of aging^3,54^. We used TTALE^telo^to monitor changes in telomere length in three established human aging systems: (1) Werner syndrome (WS) MSCs derived from WRN-deficient ESCs^7,55,56^ (Figure 6A and 6B, and Supplementary information, Figure S7A), (2) MSCs differentiated from iPSCs derived from Hutchinson-Gliford progeria syndrome (HGPS) patients (HGPS-GC-MSCs as an isogenic control line differentiated from *LMNA* gene-corrected HGPS-iPSCs)^40,43,57^ (Figure 6A and 6B, and Supplementary information, Figure S7B), and (3) hMSCs undergoing replicative senescence in culture (Figure 6A and 6B)^58^. Each aging model showed a substantial reduction in telomere length determined by genomic quantitative PCR (Supplementary information, Figure S7C) as well as a decrease in TTALE^telo^signal using SIM imaging (Figure 6C). We also demonstrated in a flow cytometry-based assay a marked decrease of fluorescence intensity of mCherry-TTALE^telo^ in WS-MSCs, relative to WT-MSCs, using co-expressed NLS-EGFP as a transfection control (Supplementary information, Figure S7D-S7E). In addition, we examined whether MSC aging is associated with the altered epigenetic status of centromeric DNA using EGFP-TTALE^centro^. TTALE^centro^ signals were more diffused and less intense in WS-specific human MSCs compared with their WT counterparts (Figure 6D-6F and Supplementary information, Movies S3 and S4). This is consistent with the reported heterochromatin disorganization at centromeres in WS MSCs^7^, which leads to active transcription from the disorganized centromeric repetitive elements (Supplementary information, Figure S7F). In WRN-deficient ESCs and NSCs used as negative controls, the telomeric length, condensation state of centromeres, and transcription from centromeric elements were not altered by WRN deficiency (Supplementary information, Figure S8A-S8F).

**Figure 7:**
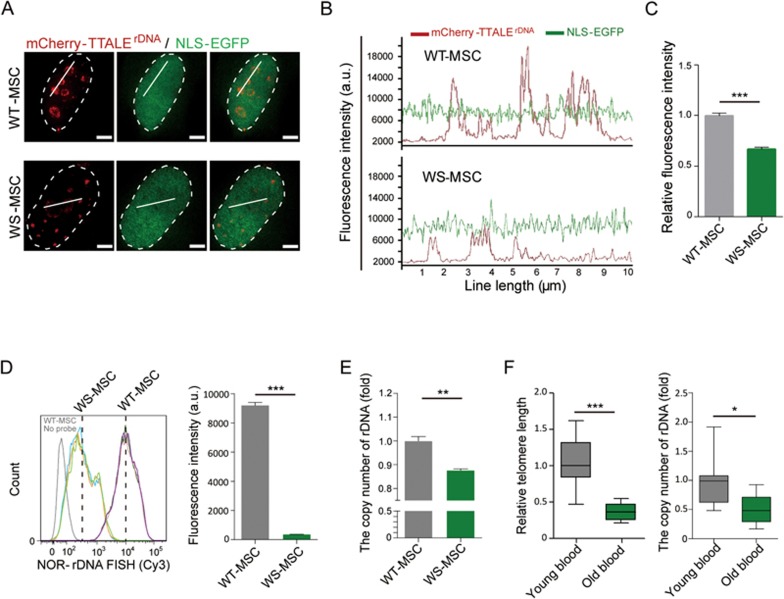
TTALE-based imaging indicating physical attrition of NOR-rDNAs in senescent WS-MSCs. **(A)** SIM images showing mCherry-TTALE^rDNA^-labeled NOR-rDNA in WS-MSCs and WT-MSCs. NLS-EGFP was co-expressed to monitor transfection efficiency. Dashed lines indicate the nuclear boundary. Scale bars, 5 μm. **(B)** Intensity profiles of TTALE^rDNA^ signals across the solid lines (10 μm in length) in **A**. **(C)** Histogram showing fluorescence intensity of mCherry-TTALE^rDNA^ normalized by that of NLS-GFP in A. n = 50 nuclei; ^***^P < 0.001. **(D)** Quantitative FISH (FACS) analysis of NOR-rDNA in WS-MSCs and WT-MSCs. Left panel: primary result of FACS. Right panel: histogram showing lower average intensity of NOR-rDNA FISH signal in WS-MSCs compared with WT-MSCs. Data are presented as mean ± SEM; *n* = 3; ^***^P < 0.001. **(E)** Quantitative PCR analysis showing diminished rDNA copy numbers in WS-MSCs relative to WT-MSCs. Data are presented as mean ± SEM; *n* = 3; ^**^P < 0.01. **(F)** qPCR analysis of of telomere length (left) and NOR-rDNA copy number (right) in the genomic DNA of peripheral blood samples collected from young and old donors. *n* = 8 (young donors) or 9 (old donors); ^***^P < 0.001; ^*^P < 0.05.

We also investigated potential changes of NOR-rDNA loci in aged human stem cells. The fluorescence intensity of mCherry-TTALE^rDNA^ was significantly lower in the nuclei of senescent WS-MSCs compared with WT-MSCs (Figure 7A-7C); a co-transfected nuclear-targeted GFP (NLS-GFP) was used as an internal control. Furthermore, flow FISH and PCR indicated that the copy number of NOR-rDNA repeats was significantly decreased in senescent WS-MSCs (Figure 7D and 7E), whereas no significant decrease was observed when the copy number of *GAPDH* locus was analyzed (Supplementary information, Figure S9A). Similar reductions of NOR-rDNA copy number and mCherry-TTALE^rDNA^ fluorescence intensity were also observed in HGPS-MSCs undergoing accelerated senescence and in replicative senescent MSCs (Supplementary information, Figure S9B-S9G). To identify whether the NOR-rDNA attrition observed during premature human MSC senescence can be extended to human physiological aging, peripheral bloods from young (6-10 years old) and old (69-72 years old) donors were obtained to detect the NOR-rDNA copy number, as well as telomere length. As expected, we observed significant shortening of telomeres in blood samples from old individuals (Figure 7F, left panel). Importantly, the copy numbers of NOR-rDNA in peripheral blood of old donors were also diminished relative to those of young donors (Figure 7F, right panel). Together, using TTALE-based imaging, we have not only validated telomere attrition and centromere disorganization in senescent human cells^7,40,43^, but also provided the strong evidence that human aging is associated with attrition of NOR-rDNA repeats.

We next investigated whether fluorescence-tagged TTALEs can be used for studying aging-associated DNA damage responses (DDRs) at telomeric and centromeric loci. WT- and WRN-deficient MSCs were transfected with mCherry-TTALE^telo^ or mCherry-TTALE^centro^ and aging-associated DDR was determined by co-localization of anti-γ-H2AX immunostaining with telomeres or centromeres. We observed significantly increased γ-H2AX signals at both telomeres and centromeres in WRN-deficient MSCs relative to WT controls (Supplementary information, Figure S10A-S10D). As a positive control, activation of DDR by treatment of WT MSCs with the DNA damage agent hydroxyurea^59^ induced a similar accumulation of γ-H2AX signals at these genomic repetitive elements (Supplementary information, Figure S10A-S10D). Collectively, these results establish TTALEs as a robust tool for the study of human aging-related biology.

### TTALE-based *in vivo* imaging to track telomere attrition in telomerase-deficient mice

**Figure 8:**
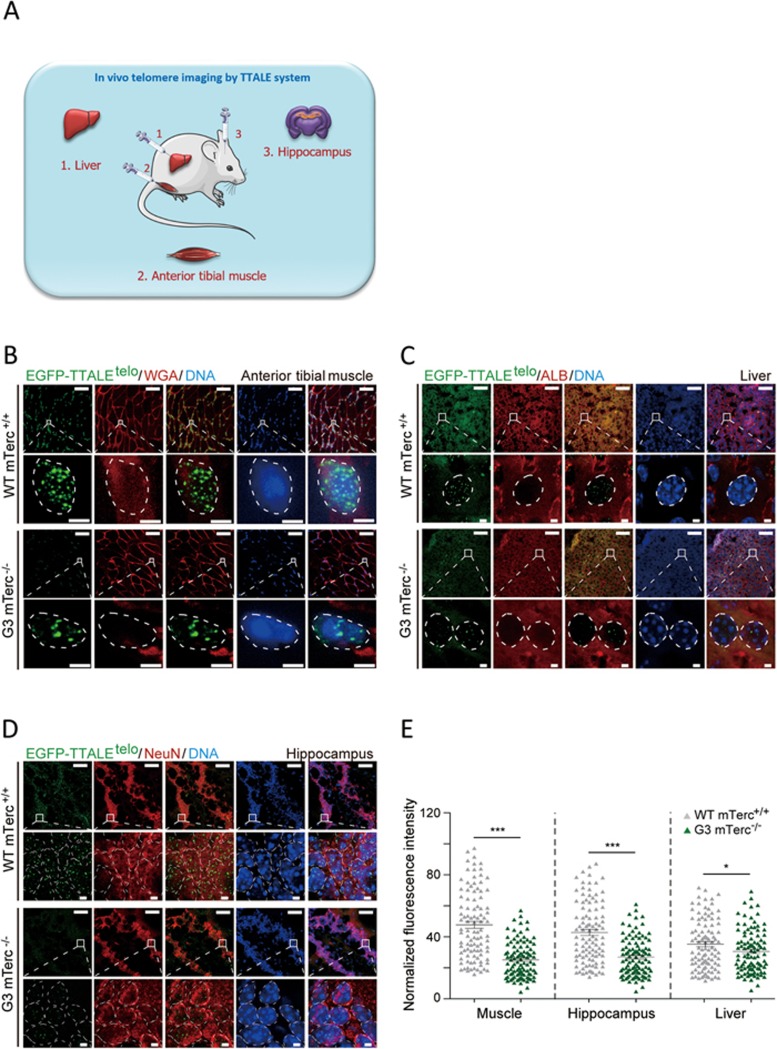
Lentiviral TTALE-mediated in situ telomere imaging in mouse tissues. **(A)** Schematic diagram of TTALE-based telomere imaging in mouse liver, anterior tibial muscle, and hippocampus. **(B-D)** Lentiviral EGFP-TTALE^telo^-mediated telomere imaging in anterior tibial muscle **(B)**, liver **(C)**, and hippocampus **(D)** in WT *mTerc^+/+^* and G3 *mTerc^−/−^* mice. Dye for wheat germ agglutinin (WGA), antibody against human serum albumin (anti-ALB), and antibody against NeuN (anti-NeuN) were used to label (in red) the outline of anterior tibial muscle cells **(B)**, the cytoplasm of liver cells **(C)**, and nuclei of the hippocampus **(D)**, respectively. Magnified images of the boxed region are shown in the lower panels. Dashed lines indicate the nuclear boundary. Scale bars, 50 μm (wide-field images) or 2 μm (magnified images). **(E)** Normalized fluorescence intensities of EGFP-TTALE^telo^signals in **B-D**. *n* = 100 nuclei; ^***^P < 0.001.

Next, we investigated whether the TTALE system can be employed to directly investigate organ and tissue aging *in vivo* in an accelerated-aging mouse model. For this purpose and as a proof of concept, we utilized previously reported telomerase-deficient mice (G3 *mTerc^−/−^*) which showed significant telomere attrition compared with WT *mTerc^+/+^* mice^60,61,62,63^ (Figure 8A and Supplementary information, Figure S11A-S11C). We developed a lentivirus-based EGFP-TTALE^telo^ system (Supplementary information, Figure S11D and S11E and Data S1), and verified its utility as a tool to precisely label telomeres in cultured human U2OS cells and mouse OP9 cells with very high efficiency (transduction efficiency is over 95%) (Supplementary information, Figure S11F-S11G). Next we delivered the purified lentiviruses directly into the hippocampus, liver, and anterior tibial muscle of WT and G3 *mTerc^−/−^* mice (Figure 8A). In each of these tissues, we observed that both telomere length (Supplementary information, Figure S11C) and EGFP-TTALE^telo^ signals (Figure 8B-8E) were markedly reduced in G3 *mTerc^−/−^* mice compared with WT mice. These results demonstrate that TTALEs can be used as a simple tool for studying tissue and organ aging *in vivo* at the single-cell level.

## Discussion

### TTALEs provide an accurate and efficient platform for imaging various human genomic elements

Here we report a novel TTALE-based live-cell imaging system that has comparable imaging quality to 3D-FISH with high signal-to-noise ratios. Using TTALE imaging, we examined various genomic loci, including repetitive sequences (telomeres, centromeres, and NOR-rDNAs) and a specific gene locus (*MUC4*). TTALE-based imaging allowed us to monitor these loci throughout the cell cycle, in particular during mitosis, which is marked by dramatic chromatin changes. Thus, TTALE represents a powerful tool for measuring key cell cycle events such as DNA replication, sister chromatid cohesion, and chromosome condensation or decondensation during mitosis. Drawing from its strengths in both specificity and flexibility, TTALE imaging offers a versatile way to monitor endogenous genomic loci, including repetitive sequences and protein-coding genes, in various cellular contexts^10^.

In particular, the TTALE system is able to label ribosomal gene loci that are organized as clusters within NORs located on the short arms of the five human acrocentric chromosomes (chromosomes 13, 14, 15, 21, and 22). NORs are implicated in protein synthesis and cell proliferation, so visualization of NOR-rDNA loci in the human genome is of great significance. Dynamic imaging of NOR-related rDNA loci has been a major challenge and has not been achieved by any existing live-cell imaging techniques. The new TTALE system thus allows, for the first time, high-resolution imaging of NOR-related ribosomal gene loci in live human cells.

### Advantages of TTALE-based imaging

The TTALE imaging system provides several advantages over existing systems for precise locus-specific genomic imaging. We demonstrated that one of the major limitations of conventional TALEs in imaging genomic repetitive sequences was unwanted formation of large aggregates, leading to mis-localization of conventional TALEs in labeled nuclei. On the one hand, conventional TALEs targeting genomic repetitive sequences may form or label some unknown subnuclear structures (perhaps different from the nucleolus-like structure formed by dCas9 proteins in the unoptimized dCas9/sgRNA system^15^). On the other hand, the formation of protein aggregates may occur due to the very high local concentration of conventional TALEs when being simultaneously tethered to multiple copies of repetitive sequences. Both possibilities warrant further investigation. The fusion of TALE with TRX effectively prevents the formation of aggregates within the nucleus, and the redox-catalytic activity of TRX is indispensable for precise targeting of TALEs to their corresponding genomic loci, which appears to support a chaperone-like role of fused TRX in enhancing protein solubility^30^.

In addition, dCas9 has a molecular weight of 160 KDa, which makes it difficult to deliver dCas9 together with sgRNA into mammalian cells in quantities needed for high-quality imaging, especially when dCas9 is fused with a fluorescent protein. Although selection of dCas9-EGFP stably expressed cell line is compatible to certain applications^15,16,50^, it is technically time-consuming and complicated. More importantly, clonal selection is not suitable for many human primary cell types (e.g., neurons and oocytes) that are resistant to clonal expansion. Also, although dCas9-based imaging has been used to monitor dynamic change in telomeres, the imaging quality was suboptimal compared with TTALE-based imaging. The relatively small size of TTALE probes allows for efficient delivery and expression, even when fused with fluorescent proteins. This advantage is especially critical for *in vivo* applications. Here we show that a lentiviral vector-based TTALE system can efficiently mediate *in situ* imaging of telomeres at single-cell levels in different mouse tissues, allowing the tracking of telomere attrition in telomerase-deficient mice^60,61,62,63^.

The TTALE system proves to be robust for live cell imaging. This is an important advantage over genomic imaging using FISH, which can only be done in fixed cells. The fluorescence-labeled TTALE allowed direct visualization of genomic loci (including NOR-rDNAs) in living cells. Indeed, using this technique, we demonstrated that rDNAs were predominantly localized at the surface of the nucleolus surrounding the nucleolar proteins fibrillarin and nucleolin, which is consistent with and complementary to the current model proposed for nucleolar organization^64^. In addition, the TTALE system enabled the evaluation of dynamic changes during chromatin reorganization at all stages of mitosis.

### TTALE allows simultaneous multicolor imaging of various genomic sequences in a single cell

Multicolor imaging of different genomic loci in single cells is critical for gaining insights into the relationships and/or cross-talk between various genomic loci during complex cellular processes such as mitosis, differentiation/reprogramming, and aging. We have shown that TTALE-based imaging allows for simultaneous visualization of multiple genomic loci (telomeres, centromeres, and NOR-related rDNAs) within a single nucleus. The dCas9/sgRNA-based system has also been utilized for multicolor imaging in live cells, but relies on delivery of different fluorescence-fused dCas9 variants from different bacterial species^65,66,67,68^. Again, given the technical challenge of introducing multiple dCas9 proteins of high-molecular weight, together with their guide RNA and transactivator partners, into the same cell at a sufficient quantity for high-quality imaging (Supplementary information, Figure S3A)^15^, the relatively simple TTALE system has obvious advantages.

### TTALE probes exhibit excellent performance in all human cells examined, irrespective of lineage, differentiation, and other factors

Imaging systems based on dCas9/sgRNA or conventional TALEs have only been tested in a limited number of human cell types^15,20^. In our current study, the TTALE system was validated in a variety of human and mouse cell types encompassing different differentiation stages, tissue origins, and cellular processes, including mitosis, oncogenic transformation, and cellular aging. Importantly, the efficacy of TTALEs has also been demonstrated in different tissues *in vivo.* On the basis of our findings, TTALE imaging constitutes a simple and generalized method for precise imaging of predetermined genomic loci. In addition, even for visualization of a single-gene locus (*MUC4*), TTALE faithfully labeled cognate sequences in both diploid (MSC) and aneuploidy (HeLa) human cells, demonstrating the sensitivity, accuracy, and reliability of this approach in different ploidy contexts. This feature may be of particular use for monitoring gene copy numbers in live cells, which can serve as an indicator for gene deletions and amplifications as well as chromosome translocations, under various physiological and pathological contexts^69^.

### TTALE imaging allows identification of novel chromatin alterations during aging

**Figure 9:**
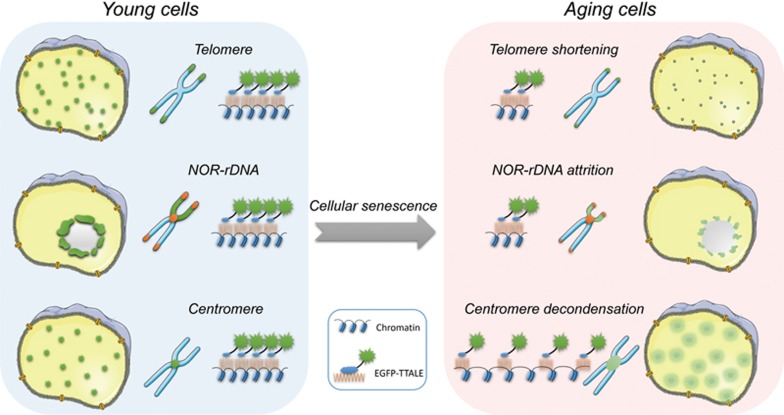
Visualization of aging-associated alterations in genomic repetitive elements by TTALE systems indicating physical attrition of telomeric DNA repeats and NOR-rDNA repeats, as well as decondensation of centromeric DNAs.

Here we report for the first time the direct visualization of heterochromatin disorganization at centromeres during human stem cell aging, further confirming our previous findings^7^. In addition, we successfully observed telomere shortening^3^ at the single-cell level both in different human stem cell models of accelerated aging disorders and *in vivo* in tissues of telomerase-deficient mice. Our most striking finding was the loss of NOR-rDNA TTALE signals in aged human cells. We identified reduction of NOR-rDNA copy number as a novel molecular hallmark of human aging. It should be noted that reduction of rDNA repeats has recently been reported during yeast aging^70,71,72^. In addition, supporting our conclusion a case report in 1979 suggested aging-associated loss of rDNA in human postmortem myocardium and brain^73,74^. On the basis of our findings, we propose a model in which human aging is associated with remodeling of genomic repetitive elements, including attrition of telomeric and NOR-rDNA repeats, as well as reorganization of centromeric DNA elements (Figure 9). It should be noted that telomeres, centromeres, and NOR-rDNAs constitute the major components of constitutive heterochromatin within the nucleus^75^. We also observed a coordinated enhancement of DDRs at telomeric and centromeric loci in human stem cell aging models. Our TTALE-based imaging results provide evidence that human aging involves a complex interplay between genetic and epigenetic instabilities facilitated by changes in 3D chromatin organization.

## Materials and Methods

### Reagents

The following antibodies were used: anti-ALB (Abcam, ab8940, 1:400); anti-β-actin (Santa Cruz Biotechnology, sc-130301, 1:5 000); anti-caldesmon (Sigma-Aldrich, C4562, 1:300); anti-CD73 (BD Biosciences, 550741, 1:50); anti-CD90 (BD Biosciences, 555595, 1:100); anti-CD105 (eBioscience, 1-1057, 1:100); anti-CENPA (Abcam, ab13939, 1:400); anti-fibrillarin (Abcam, ab4566, 1:100); anti-FLAG (Sigma-Aldrich, M2, 1:2 000 for western blotting, 1:400 for immunofluorescence); anti-GAL4 (Abcam, ab14477, 1:1 000 for western blotting, 1:400 for immunofluorescence); anti-γH2AX (Millipore, 05-636, 1:400); anti-IgG-APC (eBioscience, 555751, 1:100); anti-IgG-FITC (eBioscience, 555748, 1:100); anti-IgG-PE (eBioscience, 555749, 1:100); anti-MAP2 (Sigma-Aldrich, m4403, 1:500); anti-NANOG (Abcam, ab21624, 1:250); anti-nestin (Millipore, MAB5326, 1:500); anti-NeuN (Millipore, ABN78 1:400); anti-OCT4 (Santa Cruz Biotechnology, sc-5279, 1:100); anti-Nucleolin (Abcam, ab22758, 1:200); anti-PAX6 (Covance, PRB-278P, 1:500); anti-SMA (Sigma-Aldrich, A5228, 1:100); anti-SM22 (Abcam, ab14106, 1:200); anti-SOX2 (Santa Cruz Biotechnology, sc-17320, 1:100); anti-Tuj1 (Sigma-Aldrich, T2200, 1:500); Alexa Fluor 555-conjugated wheat germ agglutinin (Thermo Fisher, W32464, 1:500). Other reagents, including deoxycytidine, hydroxyurea, NAC, nocodazole, and thymidine, were purchased from Sigma-Aldrich.

### Plasmid and TALE construction

TALEs recognizing specific target sites were constructed using the Golden Gate Assembly method with the TALE Toolbox kit (Addgene, 1000000019)^25^. Target sequences and corresponding alignment of repeat-variable di-residues are shown in Supplementary information, Table S1. To construct the conventional TALE plasmids, FokI nuclease was removed from pTALEN-V2 backbone plasmids (Addgene, 32189, 32190, 32191, and 32192) using the In-Fusion HD Cloning kit (Clontech). To construct TALE-tag backbone plasmids, EGFP, mCherry, CFP, YFP, TRX, SUMO1, SUMO2, SUMO3, UB, MBP, GST, and GRX fusion tags were subcloned into the cloning site downstream of the conventional TALE plasmids. The coding sequences of the TRX, SUMO1, SUMO2, SUMO3, UB, MBP, GST, and GRX fusion tags were amplified from a cDNA library of H9 human ESCs, and the primer sequences for PCR are listed in Supplementary information, Table S2. PCR products were cloned into pEASY-Blunt (TransGen Biotech) for sequence verification and further cloning. The fluorescent protein-TTALE (TALE-FP-TRX) vector was generated by placing a 36-bp linker (5′-TCCGGACTCAGATCTCGAGCTCAAGCTTCGAATTCC-3′) between the fluorescent protein and TRX.

### Cell culture

U2OS, HeLa, MCF7, HepG2, and HEK293 cells (ATCC) were cultured in Dulbecco's modified Eagle Medium (DMEM; Invitrogen) supplemented with 10% fetal bovine serum (FBS, Gemini Bio-Products). H9 human ESCs (WiCell Research Institute) and iPSCs were maintained on mouse embryonic fibroblast feeder cells in human ESC culture medium containing 80% DMEM/F-12 (Invitrogen), 20% knockout serum replacement (Invitrogen), 2 mM GlutaMAX (Invitrogen), 1% non-essential amino acids (Invitrogen), 1% penicillin/streptomycin (PS, Invitrogen), 55 μM β-mercaptoethanol, 10 ng/ml human basic fibroblast growth factor (bFGF, Joint Protein Central). They were then transferred to Matrigel (BD Biosciences)-coated plates in mTeSR1 medium (STEMCELL Technologies). Human MSCs were grown on 0.1% gelatin-coated plates in MSC culture medium containing 90% MEM Alpha Medium (Invitrogen), 10% FBS (AusGeneX), 1% non-essential amino acids, 1% PS, and 1 ng/ml bFGF. Human NSCs were cultured in NSC maintenance medium containing 50% Advanced DMEM/F12 (Invitrogen), 50% Neurobasal Medium (Invitrogen), 1× N2 Supplement (Invitrogen), 1× B27 Supplement (Invitrogen), 2 mM GlutaMAX, 10 ng/ml human leukemia inhibitory factor (hLIF, Millipore), 3 μM CHIR99021 (Selleck), and 2 μM SB431542 (Selleck). Human postmitotic neurons were cultured in neural differentiation medium containing 50% Advanced DMEM/F12, 50% Neurobasal medium, 1× N2, 1× B27, 2 μM GlutaMAX, 10 ng/ml human brain-derived neurotrophic factor (Peprotech), 10 ng/ml human glial cell line-derived neurotrophic factor (Peprotech), 200 μM ascorbic acid (Sigma-Aldrich), and 400 μM dbcAMP (Sigma-Aldrich). hVSMCs were cultured in VSMC culture medium containing 50% DMEM/F12, 50% Neurobasal medium, 1× N2, 1× B27, and 10 ng/ml PDGF-BB (Peprotech).

### Differentiation and characterization of human MSCs

hMSCs were differentiated from hESCs as previously described^42^. Briefly, embryoid bodies were left to differentiate in MEM Alpha Medium (Invitrogen) supplemented with 10% FBS (AusGeneX), 10 ng/ml bFGF, 5 ng/ml transforming growth factor β (HumanZyme), and 1% PS until fibroblast-like cells were observed. The hMSC-like cells were purified by flow cytometry using antibodies against hMSC surface markers (CD73, CD90, and CD105).

### Differentiation and characterization of human NSCs

Neural induction was performed according to previous reports^8,42^. ESCs were first cultured for 2 days with NIM-1 medium containing 50% Advanced DMEM/F12 (Invitrogen), 50% Neurobasal medium (Invitrogen), 1× N2 (Invitrogen), 1× B27 (Invitrogen), 2 mM GlutaMAX (Invitrogen), 10 ng/ml hLIF (Millipore), 4 μM CHIR99021 (Selleck), 3 μM SB431542 (Selleck), 2 μM Dorsomorphin (Sigma-Aldrich), and 0.1 μM Compound E (EMD Chemicals). The culture medium was then switched to NIM-2 (NIM-1 without Dorsomorphin) for 5 days. Finally, cells were split and maintained in NSC maintenance medium.

### Differentiation and characterization of human neurons

The differentiation of neurons was performed according to previous reports^8,42^. NSCs were split onto Matrigel/laminin (Sigma-Aldrich)-coated plates at a density of 1 × 10^4^ cells/cm^2^ in neuronal differentiation medium containing DMEM/F12 (Invitrogen), 1× N2 (Invitrogen), 1× B27 (Invitrogen), 200 μM ascorbic acid (Sigma-Aldrich), 400 μM dbcAMP (Sigma-Aldrich), 10 ng/ml glial cell line-derived neurotrophic factor (Peprotech), and 10 ng/ml brain-derived neurotrophic factor (Peprotech). Cells were cultured continuously for 21 days and characterized by immunostaining.

### Derivation and characterization of human VSMCs

Human ESCs were differentiated into VSMCs as previously described^76^ with modification. Briefly, H9 hESCs were cultured on Matrigel-coated plates in mTESR1 (STEMCELL Technologies) for 24-48 h, then switched to VSMC differentiation medium containing 3 μM CHIR99021 (Cellagentech) and 25 ng/ml BMP4 (Humanzyme) for 3 days, and then to VSMC differentiation medium containing 10 ng/ml PDGF-BB (Peprotech) and 2 ng/ml activin A (Humanzyme) for another 2 days. The differentiated cells were then cultured continuously in VSMC culture medium for up to 10 passages. At passage 3, VSMCs were characterized and used for experiments.

### Reprogramming of human NSCs to iPSCs

NSCs were reprogrammed with episomal vectors expressing OCT4, SOX2, KLF4, Lin28, and L-MYC following a previously described protocol^77,78^. The iPSC lines generated were maintained in human ESC culture medium (above) on mouse embryonic fibroblast feeder cells or in mTeSR1 medium (STEMCELL Technologies) on Matrigel.

### Transient transfection

Different approaches of transient transfection were performed in accordance with the cell type, including electroporation (hESC), FuGene HD (Promega; hNSC), Lipofectamine 3000 (Invitrogen; human postmitotic neuron, hMSC, hVSMC, HEK293, HeLa, U2OS, MCF7, HepG2). Transfection was performed when cells were 70% - 80% confluent using a total of 100 ng plasmid per well in 24-well plates. At 48-72 h after transfection, cells were fixed or harvested for subsequent experiments.

### Immunofluorescence

Cells seeded on coverslips (Fisher) were fixed in 4% paraformaldehyde (PFA) for 15 min, permeabilized with 0.4% Triton X-100 in PBS for 10 min, and blocked with 10% donkey serum in PBS (Jackson ImmunoResearch) for 1 h. Cells were then incubated with primary antibody in the blocking buffer at 4 °C overnight, washed with PBS three times, incubated with secondary antibody at room temperature for 1 h, washed again with PBS three times, and counterstained with Hoechst 33342 (Invitrogen). Quantitative microscopy was performed using randomly chosen cells for each sample^15^. The number, intensity, and area of fluorescent signals were analyzed using Image J software (NIH).

### 3D-FISH/ immunolabeling

Cy3-labeled telomere and centromere PNA FISH probes were purchased from Panagene. Cy3-labeled PNA probe targeting 28S rDNA was synthesized by Panagene and the probe sequence is the same as the target sequence of TTALE^rDNA^. PNA FISH was combined with immunostaining to detect telomeres and centromeres. Cells were permeabilized with 0.4% Triton X-100 at room temperature for 10 min, incubated with RNase A (100 μg/ml in PBS) at 37 °C for 30 min, blocked with 10% donkey serum in PBS for 30 min, and treated with streptavidin solution (four drops streptavidin blocking buffer (Vector Laboratories) in 1 ml PBST (PBS with 0.2% (v/v) Tween-20)) for 15 min at room temperature. Cells were then incubated with primary antibody in biotin solution (four drops biotin blocking buffer (Vector Laboratories) in 1 ml PBST) and subsequently biotin-labeled secondary antibody (Vector Laboratories) in PBST. The cells were then washed with PBST three times (each for 5 min) and transferred into 20% glycerol/PBS for 30-60 min at room temperature. After that, cells were heated at 85 °C for 15 min in hybridization mixture (70% formamide, 1% blocking buffer, 10 mM Tris-HCl, pH 7.2, 0.5 mg/ml Cy3-labeled PNA probe), and then placed in a dark humidified chamber at room temperature for 2-3 days. After hybridization, cells were washed three times (each for 10 min) with preheated 2× SSC at 55-60 °C, once with 2× SSC at room temperature for 2 min, and once with 1× SSC at room temperature for 2 min. Hybridized cells were incubated with Alexa Fluor 488-conjugated streptavidin (Vector Laboratories) diluted in the blocking solution (4× SSC with 0.2% Tween-20 and 10% donkey serum) at room temperature for 45 min in the dark humidified chamber, washed twice for 5 min in 4× SSC/Tween-20 at 37 °C, and stained with Hoechst 33342 (Invitrogen).

### Optical setup and image acquisition

Confocal images of all human cells were acquired on the TCS SP5 II laser scanning confocal imaging system (Leica) with an HC PL APO 40×/0.85 NA CORR CS objective (Leica) or HC PL APO 63× 1.40-0.60 NA oil-immersion objective (Leica) and Multispectral Ar ion lasers (405 diode for Hoechst, argon for EGFP or Alexa 488, and DPSS 561 for mCherry, Alexa 568, or Cy3) in conjunction with LAS AF 2.2 software (Leica). To reduce photobleaching, a low-excitation laser power density was chosen by setting the pinhole size as 1 Airy. Samples were imaged with a resolution of 512 × 512, scanning speed of 400 Hz, and line average mode. The digital magnification was 12-fold for imaging chromosomes during mitosis and 9-fold for all other samples. Serial *z*-stack sectioning was done at 0.5-μm intervals for all samples. Regular image processing was performed using Photoshop CS5 software (Adobe). Fluorescence intensity and the number and area of fluorescent signals were measured using ImageJ software (NIH).

### 3D-SIM super-resolution microscopy and image analysis

All 3D-SIM images of human cells were acquired on the DeltaVision OMX V3 imaging system (GE Healthcare) with a 100× 1.4 NA oil-immersion objective (Olympus, UPlanSApo), solid-state multimode lasers (488, 405, and 561 nm), and electron-multiplying charge-coupled device cameras (Evolve 512×512, Photometrics). Serial *z*-stack sectioning was done at 125-nm intervals for SIM mode. The microscope was routinely calibrated with 100-nm fluorescent spheres to calculate both the lateral and axial limits of image resolution. SIM image stacks were reconstructed using SoftWoRx 6.1.1 (GE Healthcare) with the following settings: pixel size, 39.5 nm; channel-specific optical transfer functions; Wiener filter constant, 0.0010; discard negative intensities background; drift correction with respect to first angle; and custom K0 guess angles for camera positions. The reconstructed images were further processed for maximum-intensity projections with SoftWoRx 6.1.1. Pixel registration was corrected to be < 1 pixel for all channels using 100-nm Tetraspeck beads. Images in Figures 4C, 4D, 5B, 6B, 6D, 7A and Supplementary information, Figures S3B, S3C, S8A-D, S9B and S9E are 3D-SIM images acquired on the DeltaVision OMX V3 imaging system (GE Healthcare).

### SIM-TEM

**Preparation of support film** For fine correlation between light and transmission electron microscopy (LM and TEM), we deposited 200-nm blue fluorescent beads (Invitrogen; 1:400 dilution) on the surface of 200-mesh EM-finder copper grids (Gilder Grids) coated with carbon film before applying to resin sections.

**Sample preparation for correlative LM and TEM** Transfected HEK293 cells were harvested enzymatically by trypsin and fixed in 4% PFA in PBS, pH 7.4, on ice for 2 h. Samples were dehydrated in a graded series of ethanol and then infiltrated and embedded in Lowicryl resin HM20. Embedded samples were sliced into 200-nm sections using a Leica ultramicrotome EM UC6 (Leica) and sections were collected on the prepared film.

**3D-SIM** Images of all human cell types were acquired on the DeltaVision OMX V3 imaging system (GE Healthcare) with a 100× 1.4-NA oil-immersion objective (Olympus UPlanSApo), solid-state multimode lasers (488, 405, and 561 nm) and electron-multiplying charge-coupled device cameras (Evolve 512512, Photometrics). Serial *z*-stack sectioning was done at 250-nm intervals for conventional mode. The microscope was routinely calibrated with 100-nm fluorescent spheres to calculate both the lateral and axial limits of image resolution. Conventional image stacks were processed by deconvolution methods using SoftWoRx 5.0 (GE Healthcare) with Wiener filter enhancement and Wiener filter smoothing each set at 0.900. The reconstructed images were further processed for maximum-intensity projections with SoftWoRx 6.1.1. Pixel registration was corrected to be < 1 pixel for all channels using 100-nm Tetraspeck beads.

**TEM imaging** After fluorescence imaging, TEM sample grids were transferred and imaged under a Spirit Transmission Electron Microscope (FEI Company) operating at 100 kV.

**Alignment of LM with EM** Currently, two methods have been introduced to correlate LM images with EM images. The first method utilizes fiducial markers, whereas the second uses the image details from both LM and EM. Here, we applied the first method for alignment of 2D LM images with a single TEM projection image. We used electron-dense fluorescent beads, which were visible under both LM and EM. The fluorescent beads in the blue channel of LM images were used for registration between fluorescent signals and low-magnification electron micrographs. The localization of beads in LM was performed by least square curve fitting of 2D Gaussian distributions, and the localization of beads in EM was performed with 2D top-hat distributions. During correlative imaging, we localized the beads in both LM and EM images, and calculated the transformation between the two point sets. Finally, we applied the transformation matrix to the LM images. The registration precision could be assessed by calculating the position error of the beads between the LM and EM images.

### Human oocyte microinjection and live-cell imaging

Human germinal vesicle (GV) oocytes were donated by infertile patients in intracytoplasmic sperm injection cycles because of male factors. Excess human GV oocytes were donated by infertile patients following collections for intracytoplasmic sperm injection due to male infertility. The donation procedure was approved by the Institutional Review Board of Peking University Third Hospital and all oocyte donors voluntarily provided informed consent. The human GV oocytes were transferred into G-MOPES medium (Vitrolife) and the desired vector (1 μg/μl) was directly injected into the cytoplasm using a FemtoJet microinjector (Eppendorf). Successfully injected oocytes were quickly transferred to a culture chamber with maturation medium (Origio) for live oocyte imaging on an UltraVIEW VoX confocal imaging system (PerkinElmer) using a 20× objective and a 488-nm laser for green fluorescence detection.

### Synchronization of HeLa cells

For TTALE imaging of HeLa cells at different stages of mitosis, cells were treated for cell cycle arrest as previously described^79^. In brief, 24 h after transfection of TTALE vectors, cells were treated with 2 mM thymidine for 14 h. After washing with pre-warmed PBS, cells were cultured in growth medium supplemented with 24 μM deoxycytidine for 9 h followed by a second treatment of thymidine for another 14 h. After release from thymidine block for 2 h, nocodazole (0.1 μg/ml) was used for 10 h to induce synchronization from mitosis. Subsequently, cells were harvested for immunofluorescence or FISH/immunofluorescence at different stages of mitosis.

### Real-time quantitative PCR

Total cellular RNA was extracted using TRIzol (Invitrogen), genomic DNA was removed using a DNA-free kit (Ambion), and cDNA synthesis was carried out using the GoScript Reverse Transcription system (Promega). Real-time quantitative PCR (qPCR) was performed using iTaq Universal SYBR Green Supermix (Bio-Rad) on a CFX384 Real-Time PCR system (Bio-Rad). All primers for real-time qPCR are listed in Supplementary information, Table S3.

### GFP intensity measurement by flow cytometry

Cells were collected 24, 48, 72, 96, or 120 h after transfection and washed with PBS one time. GFP intensity was measured as cell counts using a LSRFortessa cell analyzer (BD Biosciences) with the FITC-A channel. Data were analyzed using FlowJo software (BD Biosciences).

### Western blotting

Cells were lysed in RIPA buffer (25 mM Tris-HCl, pH 7.6, 150 mM NaCl, 1% Nonidet P-40, 1% sodium deoxycholate, 0.1% SDS) with protease inhibitor cocktail (Roche). Protein quantification was performed using a BCA kit. Protein lysate (∼20 μg) was subjected to SDS-PAGE and transferred to a PVDF membrane (Millipore). Primary- and HRP-conjugated secondary antibodies were used to visualize target proteins. Image acquisition and quantification of western blot signals were performed with Image Lab software of the ChemiDoc XRS system (Bio-Rad).

### Determination of telomere length

Telomere length was analyzed as previously described^7^. In brief, genomic DNA was isolated from human cells using the DNeasy Blood & Tissue kit (Qiagen) and from mouse tissues using the MiniBEST Universal Genomic DNA Extraction kit (Takara). qPCR was performed using iTaq Universal SYBR Green Supermix (Bio-Rad) on a CFX384 Real-Time PCR system (Bio-Rad). qPCR primers are listed in Supplementary information, Table S3.

### TTALE-based measurement of telomere length by flow cytometry

Forty-eight hours after co-transfection with both NLS-EGFP and mCherry-TTALE^telo^ expression vectors, WT-MSCs and WS-MSCs were harvested and then fixed in 4% PFA for 15 min. Cells were then incubated with Hoechst (1:4 000) in PBS for 15 min, and resuspended in PBS. EGFP and mCherry dual-labeled cells were isolated by using a LSRFortessa cell analyzer (BD Biosciences) with the FITC and PE channels simultaneously. Dual-labeled cells were subsequently measured as cell counts with Pacific blue channel for identifying the proportion of Hoechst-labeled diploid cells, and with PE or FITC channel for indicating the fluorescence intensity of mCherry-TTALE^telo^ or NLS-EGFP in either WT-MSCs or WS-MSCs. FACS data were analyzed using FlowJo software (BD Biosciences). Average intensity of EGFP or mCherry from each sample was used for statistical analysis with GraphPad Prism software.

### Determination of rDNA or GAPDH copy number

rDNA or *GAPDH* copy number was analyzed by qPCR. In brief, genomic DNA was isolated from human cells using the DNeasy Blood & Tissue kit (Qiagen). A partial sequence of 28S rDNA or *GAPDH* in human genomic DNA was amplified by PCR and then cloned into pEASY-Blunt vector (TransGen Biotech). Primer sequences for PCR are listed in Supplementary information, Table S2. Following quantification, a 10-fold dilution of pEASY-Blunt-28S or pEASY-Blunt-GAPDH plasmid was used as standard samples for producing a standard curve. qPCR was performed using iTaq Universal SYBR Green Supermix (Bio-Rad) on a CFX384 Real-Time PCR system (Bio-Rad). qPCR primers are listed in Supplementary information, Table S3. The copy numbers were calculated according to the standard curve and the amount of DNA used as template for qPCR.

### Quantitative FISH of NOR-rDNA by flow cytometry

Cy3-labeled PNA probe targeting 28S rDNA was synthesized by Panagene. The probe sequence targeting 28S rDNA is the same as the target sequence of TTALE^rDNA^. Cells were harvested and then fixed in 4% PFA for 15 min, permeabilized with 0.4% Triton X-100 in PBS for 15 min, incubated with RNase A (100 μg/ml in PBS) at 37 °C for 30 min, and treated with 20% glycerol in PBS for 1 h. After that, cells were heated at 85 °C for 10 min in hybridization mixture (70% formamide, 1% blocking buffer, 10 mM Tris-HCl, pH 7.2, 0.5 mg/ml Cy3-labeled PNA probe), and then placed in a dark humidified chamber at room temperature for 2-3 h. After hybridization, cells were washed three times (each for 10 min) with preheated 2× SSC at 55-60 °C, once with 2× SSC at room temperature for 2 min, and once with 1× SSC at room temperature for 2 min. Cy3-PNA probe-labeled cells were finally measured as cell counts using a LSRFortessa cell analyzer (BD Biosciences) with the PE channel. Data of FACS were analyzed using FlowJo software (BD Biosciences). Average intensity of FISH signal from every sample was used for statistical analysis by GraphPad Prism software.

### Lentivirus production

To generate lentiviral vectors, EGFP-TTALE cassettes were inserted into the modified pEASY-Blunt Simple vector (TransGen Biotech) using *Sac*I and *Asc*I, and finally cloned into the pLE4 lentiviral vector (a kind gift from Dr Tomoaki Hishida) using *Bam*HI and *Mlu*I. To package lentivirus constructs, HEK293T cells were transfected with lentiviral vectors together with the packaging plasmids pMD2.G (Addgene, 12260) and psPAX2 (Addgene, 12259). Supernatants containing viruses were harvested at 48 and 72 h after transfection, filtered with a 0.45-μm PVDF membrane (Millipore), concentrated by ultracentrifugation at 19 400× *g* for 2.5 h, and assessed for viral titers.

### Animals

WT and homozygous Terc-null mice (*mTerc^−/−^*)^60,61,62,63^ were from the same genetic background (C57BL/6J) and were obtained from the Animal Experimental Center of Hangzhou Normal University. Generation 3 (G3 *mTerc^−/−^*) mice were generated by mating Generation 1 (G1 *mTerc^−/−^*) mice to each other and mating their offspring to each other. WT and G3 mTerc-null mice were used at 7-month-old for TTALE-mediated *in vivo* telomere imaging. Animals were housed in specific-pathogen-free conditions, bred, and maintained at the Animal Experimental Center, Institute of Biophysics, Chinese Academy of Sciences, Beijing. All animal experiments were approved by the Animal Care and Ethics Committee of Institute of Institute of Biophysics, Chinese Academy of Sciences.

### Genotyping of *mTerc^+/+^* (WT) and G3 *mTerc^−/−^* mice

Genomic DNA was isolated from mouse tissues using the MiniBEST Universal Genomic DNA Extraction kit (Takara). PCR was performed using PrimeSTAR HS DNA Polymerase (Takara). PCR primers including mTRR, 5PPgK, and mTRWtF are listed in Supplementary information, Table S2.

### *In vivo* telomere imaging

Purified lentiviruses were injected into the hippocampus of brain, liver, and anterior tibial muscle of WT *mTerc^+/+^* and G3 *mTerc^−/−^* mice. Ten days post injection, mice were killed and the brain, liver, and anterior tibial muscle were isolated and fixed with 4% PFA. Following dehydration with 30% sucrose, frozen sectioning was performed on all tissues. After immunofluorescence staining with tissue-specific markers and DNA staining with Hoechst 33342, imaging was carried out using the TCS SP5 II laser scanning confocal imaging system (Leica) with an HC PL APO 63× 1.40-0.60 NA oil-immersion objective (Leica), Multispectral Ar ion lasers (405 diode for Hoechst, argon for EGFP, and DPSS 561 for Alexa 568), and LAS AF 2.2 software (Leica). Fixed samples were imaged with a resolution of 512 × 512, scanning speed of 400 Hz, and line averaging. A digital magnification of 9-fold was used for all tissue samples, and serial *z*-stack sectioning was done at 0.5-μm intervals. Regular image processing was performed with Photoshop CS5 software (Adobe), and fluorescence intensities of whole nuclei were measured using ImageJ software (NIH).

### Statistical analysis

All data were statistically analyzed using PRISM version 5 software (GraphPad Software). Results were presented as mean ± SEM. Comparisons were conducted using the two-tailed Student's *t*-test. *P*-values < 0.05 were considered statistically significant (^*^), *P*-values < 0.01 were considered highly statistically significant (^**^) and *P*-values < 0.001 were considered highly statistically significant (^***^).

## Author Contributions

RR and LD performed the majority of the experiments. RR, LD, and KS performed the plasmid and TALE construction. RR and LD performed cell culture, differentiation, reprogramming, and TTALE-based live-cell imaging. WZ, SW, XR, and LG performed cell culture and differentiation. TX, YX, and HL performed SIM-TEM. JQ and YY performed human oocyte microinjection and live-cell imaging. ZJ, FY, RR, and LD performed animal experiments. JW, LS, XG, HT, WZ, and JL performed data analysis and wrote the manuscript. GHL, JQ, TX, RR, LD, YX, and KS conceived this study and wrote the manuscript.

## Competing Financial Interests

The authors declare no competing financial interests.

## Supplementary Material

Supplementary information, Figure S1 Unmodified TALEs were insufficient to visualize centromeres in four tumor cell lines.

Supplementary information, Figure S2 Fusion with thioredoxin relocated TALE to telomere and centromere loci in a redox-activity dependent manner.

Supplementary information, Figure S3 Comparison of TTALE and dCas9/sgRNA-based genomic imaging techniques.

Supplementary information, Figure S4 TTALE-based imaging of telomeres and centromeres during mitosis and in different human cell types.

Supplementary information, Figure S5 Characterization of human pluripotent stem cells and their differentiated derivatives.

Supplementary information, Figure S6 TTALE-based imaging of NOR-rDNAs and *MUC4*.

Supplementary information, Figure S7 TTALE-mediated visualization of telomeric and centromeric changes in human stem cell aging models.

Supplementary information, Figure S8 TTALE-mediated visualization of telomeres and centromeres in WRN-deficient ESCs and NSCs.

Supplementary information, Figure S9 TTALE-based imaging of attrition of NOR-rDNAs in senescenth MSCs.

Supplementary information, Figure S10 TTALE-mediated visualization of aging-associated DDRs at telomeric and centromeric loci.

Supplementary information, Figure S11 Lentiviral TTALE^telo^-mediated *in vivo* imaging of telomeres in telomerase-deficient mice.

Supplementary information, Table S1 Repeat variable diresidues of TALEs recognizing telomeres, centromeres, 28S rDNA, and the MUC4 locus.

Supplementary information, Table S2 PCR primer pairs and PNA-FISH probes used for vector construction, flow FISH and genotyping of gene-knockout mice.

Supplementary information, Table S3 qPCR primer pairs used for detection of centromeric and telomeric transcripts or rDNA copy number.

Supplementary information, Data S1 DNA sequencing of Lenti-EGFP-TTALEtelo construct demonstrating no recombination.

Supplementary information, Movie S1 TTALE-based live cell imaging of telomeres and centromeres in mitotic HeLa cells cotransfected with EGFP-TTALE^centro^ (green) and mCherry-TTALE^telo^ (red)

Supplementary information, Movie S2 TTALE-based live cell imaging of telomeres in GV stage-human oocytes microinjected with EGFP-TTALE^telo^ plasmid (green).

Supplementary information, Movie S3 TTALE-based imaging of centromeres in WT-MSCs. Green: EGFP-TTALE^centro^; blue: Hoechst.

Supplementary information, Movie S4 TTALE-based imaging of centromeres in WS-MSCs. Green: EGFP-TTALE^centro^; blue: Hoechst.
