# Supplementary material for: Visualization of aging-associated chromatin alterations with an engineered TALE system
Source: Cell Res. 2017 Jan 31;27(4):483–504. doi: 10.1038/cr.2017.18 (PMC5385610; doi:10.1038/cr.2017.18)
Supplement: Supplementary information, Figure S1 — Unmodified TALEs were insufficient to visualize centromeres in four tumor cell lines. [file cr201718x1.pdf]

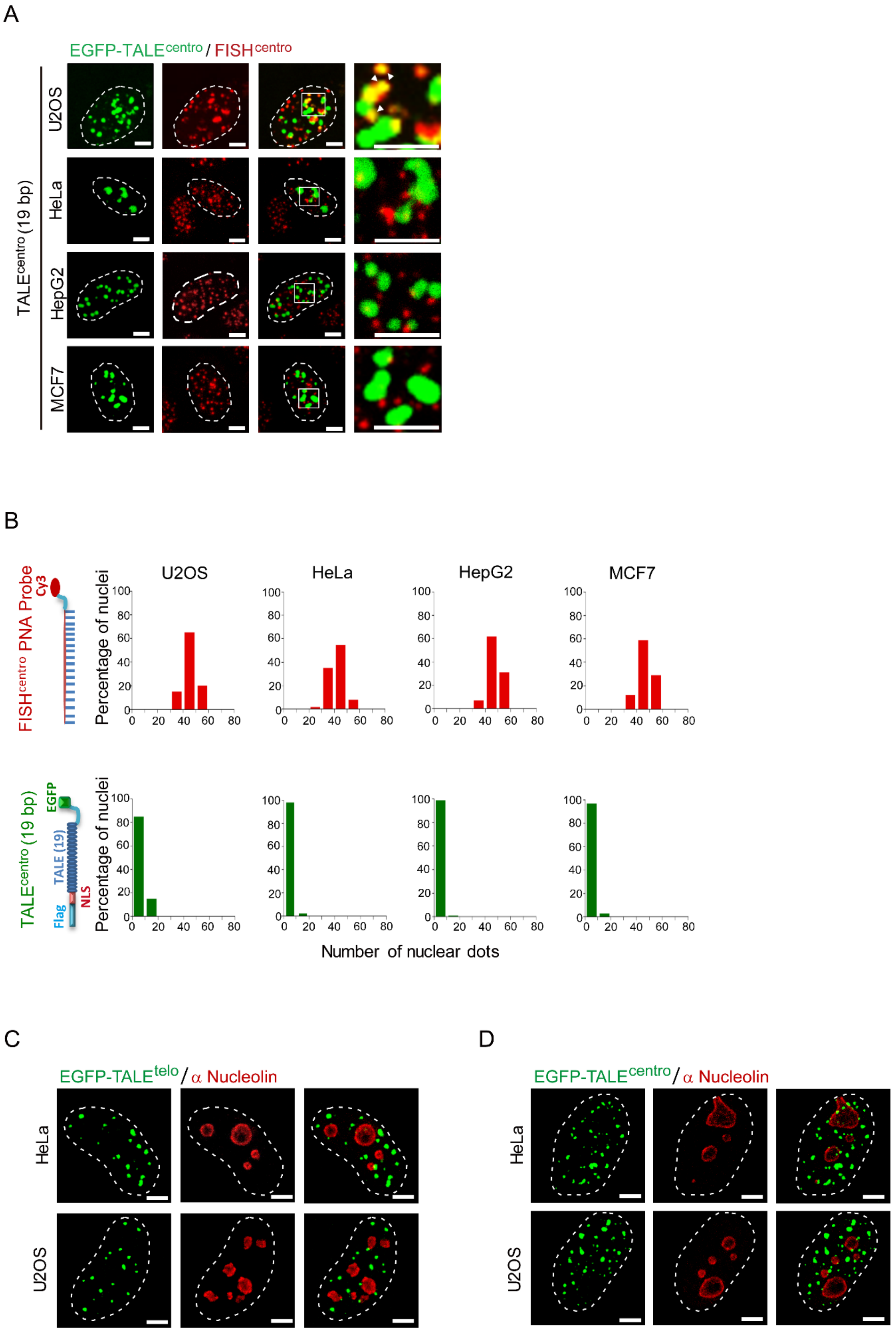

Supplementary information, Figure S1 Unmodified TALEs were insufficient to visualize centromeres in four tumor cell lines. **(A)** Co-localization analysis of EGFP-TALE^{centro} (green) and centromeric FISH (red) signals in U2OS, HeLa, HepG2, and MCF7 cells. EGFP-TALE^{centro} was designed using a 19-bp centromeric DNA sequence. Dashed lines indicate the nuclear boundary; arrowheads indicate overlapping signals. Scale bars, 5 μm. **(B)** Histograms showing numbers of centromeric FISH- or EGFP-TALE^{centro} (19 bp)-positive dots in nuclei of indicated cell lines. n = 50 nuclei per line. **(C)** The absence of co-localization between EGFP-TALE^{telo} (green) and nucleolin (red, IF) in U2OS and HeLa cells, respectively. Dashed lines indicate the nuclear boundary. Scale bars, 5 μm. **(D)** The absence of co-localization between EGFP-TALE^{centro} (green) and nucleolin (red, IF) in U2OS and HeLa cells, respectively. Dashed lines indicate the nuclear boundary. Scale bars, 5 μm.
