# Supplementary material for: Visualization of aging-associated chromatin alterations with an engineered TALE system
Source: Cell Res. 2017 Jan 31;27(4):483–504. doi: 10.1038/cr.2017.18 (PMC5385610; doi:10.1038/cr.2017.18)
Supplement: Supplementary information, Figure S2 — Fusion with thioredoxin relocated TALE to telomere and centromere loci in a redox-activity dependent manner. [file cr201718x2.pdf]

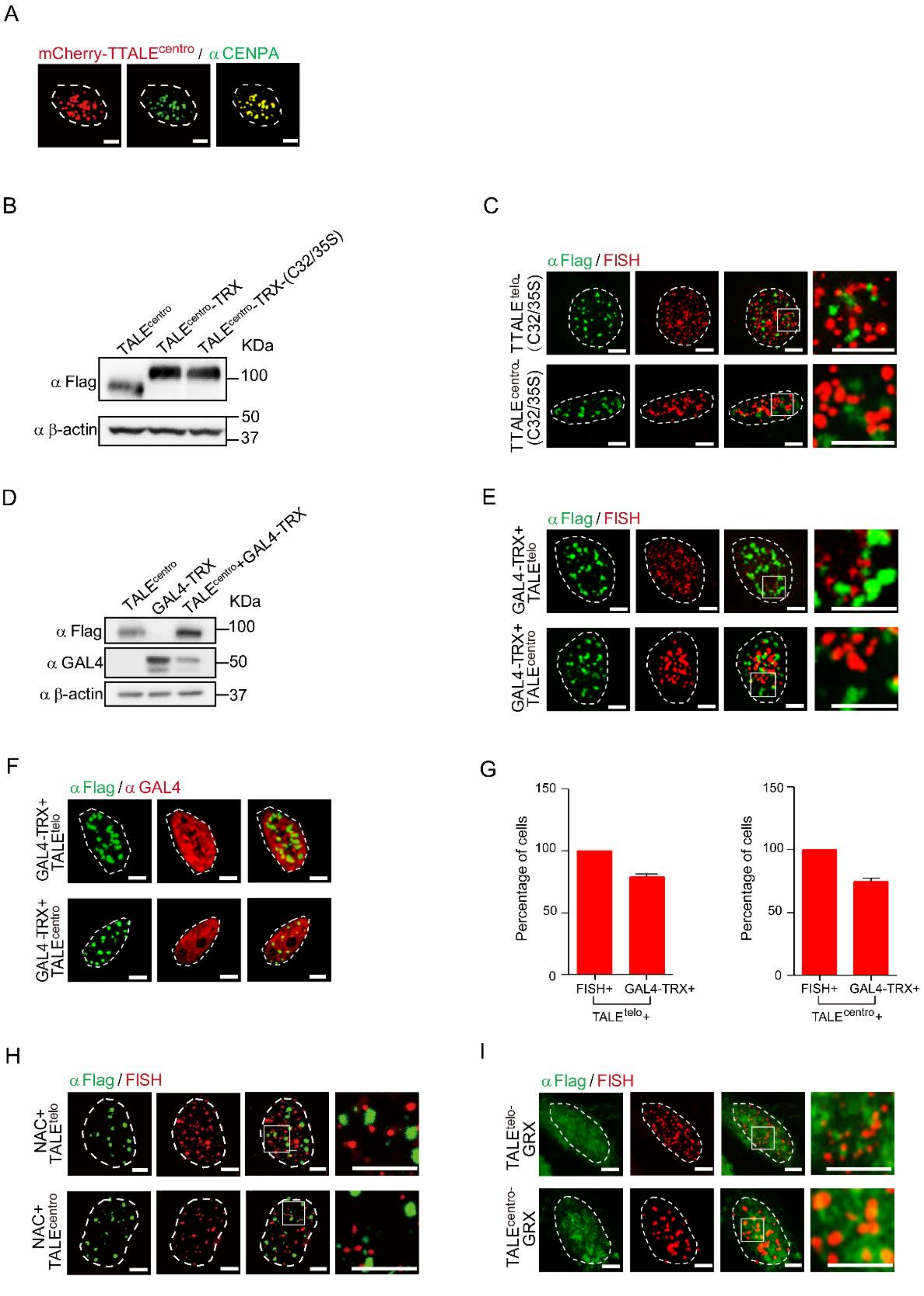

Supplementary information, Figure S2 Fusion with thioredoxin relocated TALE to telomere and centromere loci in a redox-activity dependent manner. **(A)** Co-localization of CENPA (green) and mCherry-TTALE^{centro} (red) in HeLa cells. Dashed lines indicate the nuclear boundary. Scale bars, 5 μm. **(B)** Western blot detection of TALE^{centro}, TRX-fused TALE^{centro} (TTALE^{centro}), and a redox-inactive thioredoxin mutant–fused TALE^{centro} (TRX (C32/35S)-TTALE^{centro}) in HeLa cells transiently transfected with the corresponding plasmids. β-actin was used as a loading control. **(C)** Fusion of TRX (C32/35S) failed to direct TALE to telomeric or centromeric loci. Dashed lines indicate the nuclear boundary. Scale bars, 5 μm. **(D)** Western blot detection of TALE^{centro} and GAL4-TRX in HeLa cells co-transfected with the indicated plasmids. β-actin was used as a loading control. **(E-F)** Co-localization analysis of FISH (red; E) and Flag-TALE (green; E-F) signals in HeLa cells co-expressing GAL4-TRX and Flag-TALE^{telo} or Flag-TALE^{centro}. GAL4-TRX and Flag-TALE were detected by immunofluorescence using anti-GAL4 and anti-Flag antibodies, respectively. Dashed lines indicate the nuclear boundary. Scale bars, 5 μm. No co-localization between TALE and FISH signals was observed in the presence of co-expressed GAL4-TRX (E). **(G)** Histograms showing numbers of telomeric FISH or centromeric FISH positive- and GAL4-TRX -positive cells in the pools of TALE^{telo} positive- (left) or TALE^{centro} positive- (right) HeLa cells. n = 200 cells. This result indicated that the majority of cells expressed both TALE and GAL4-TRX in Figure S2E-F. **(H)** Co-localization analysis of telomeric or centromeric FISH (red) and Flag-TALE^{telo} or Flag-TALE^{centro} (green) in HeLa cells treated with *N*-acetyl cysteine (NAC, 2.5 μM) for 24 hours. Dashed lines indicate the nuclear boundary. Scale bars, 5 μm. **(I)** Co-localization analysis of telomeric or centromeric FISH (red) and Flag-TALE^{telo} or Flag-TALE^{centro} (green) fused with glutaredoxin (GRX) in HeLa cells. Dashed lines indicate the nuclear boundary. Scale bars, 5 μm.
