# Supplementary material for: Visualization of aging-associated chromatin alterations with an engineered TALE system
Source: Cell Res. 2017 Jan 31;27(4):483–504. doi: 10.1038/cr.2017.18 (PMC5385610; doi:10.1038/cr.2017.18)
Supplement: Supplementary information, Figure S3 — Comparison of TTALE and dCas9/sgRNA-based genomic imaging techniques. [file cr201718x3.pdf]

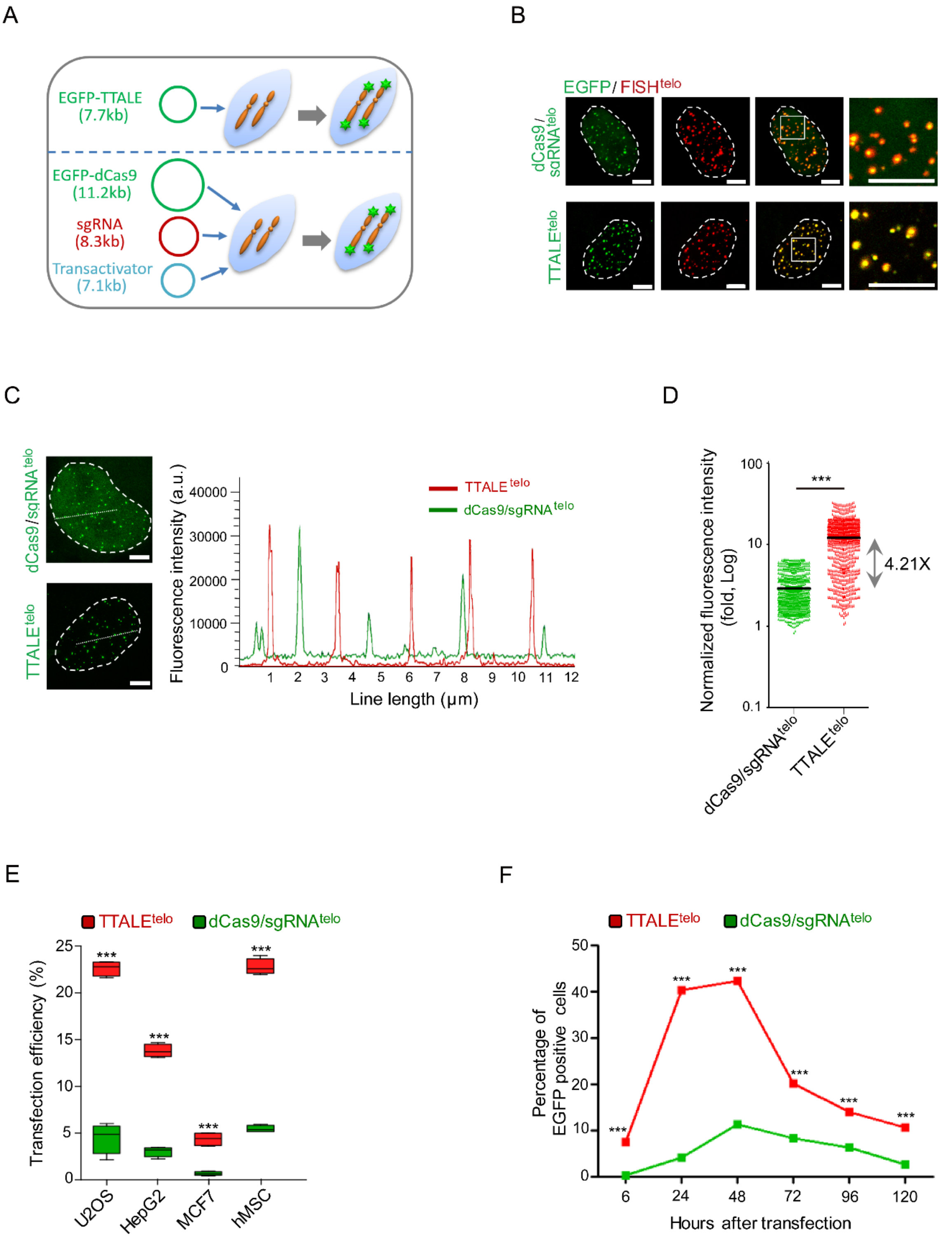

Supplementary information, Figure S3 Comparison of TTALE and dCas9/sgRNA-based genomic imaging techniques. **(A)** Schematic diagram showing different transfection complexes required for TTALE^{telo} and dCas9/sgRNA^{telo} imaging systems. **(B)** Comparison of EGFP-dCas9/sgRNA^{telo}-mediated (top) and EGFP-TTALE^{telo}-mediated (bottom) telomeric labeling. Dashed lines indicate the nuclear boundary. Scale bars, 5 μm. **(C)** Left: Telomeric imaging using EGFP-dCas9/sgRNA^{telo} (top) and EGFP-TTALE^{telo} (bottom) by SIM. Dashed lines indicate the nuclear boundary. Scale bars, 5 μm. Dotted lines represent 12 μm in length. Right: Intensity profiles across EGFP-TTALE^{telo}- and EGFP-dCas9/sgRNA^{telo}-labeled telomeres as indicated by the dotted line in the images on the left. **(D)** Log-scale scatter plot showing normalized fluorescence intensity from each identified telomere in 50 nuclei labeled by EGFP-TTALE^{telo} or EGFP-dCas9/sgRNA^{telo}. **(E)** Transfection efficiency of EGFP-TTALE^{telo} and EGFP-dCas9/sgRNA^{telo} systems detected by flow cytometry in the indicated cell types. Flow cytometry analysis was performed 24 hours post-transfection. n = 6; ***$p < 0.001$. **(F)** Time course showing transfection efficiency with dCas9/sgRNA^{telo} or TTALE^{telo} detected by flow cytometry in U2OS cells. Flow cytometry analysis was performed 6, 24, 48, 72, 96, and 120 hours after transfection. n = 6; ***$p < 0.001$.
