# Supplementary material for: Visualization of aging-associated chromatin alterations with an engineered TALE system
Source: Cell Res. 2017 Jan 31;27(4):483–504. doi: 10.1038/cr.2017.18 (PMC5385610; doi:10.1038/cr.2017.18)
Supplement: Supplementary information, Figure S4 — TTALE-based imaging of telomeres and centromeres during mitosis and in different human cell types. [file cr201718x4.pdf]

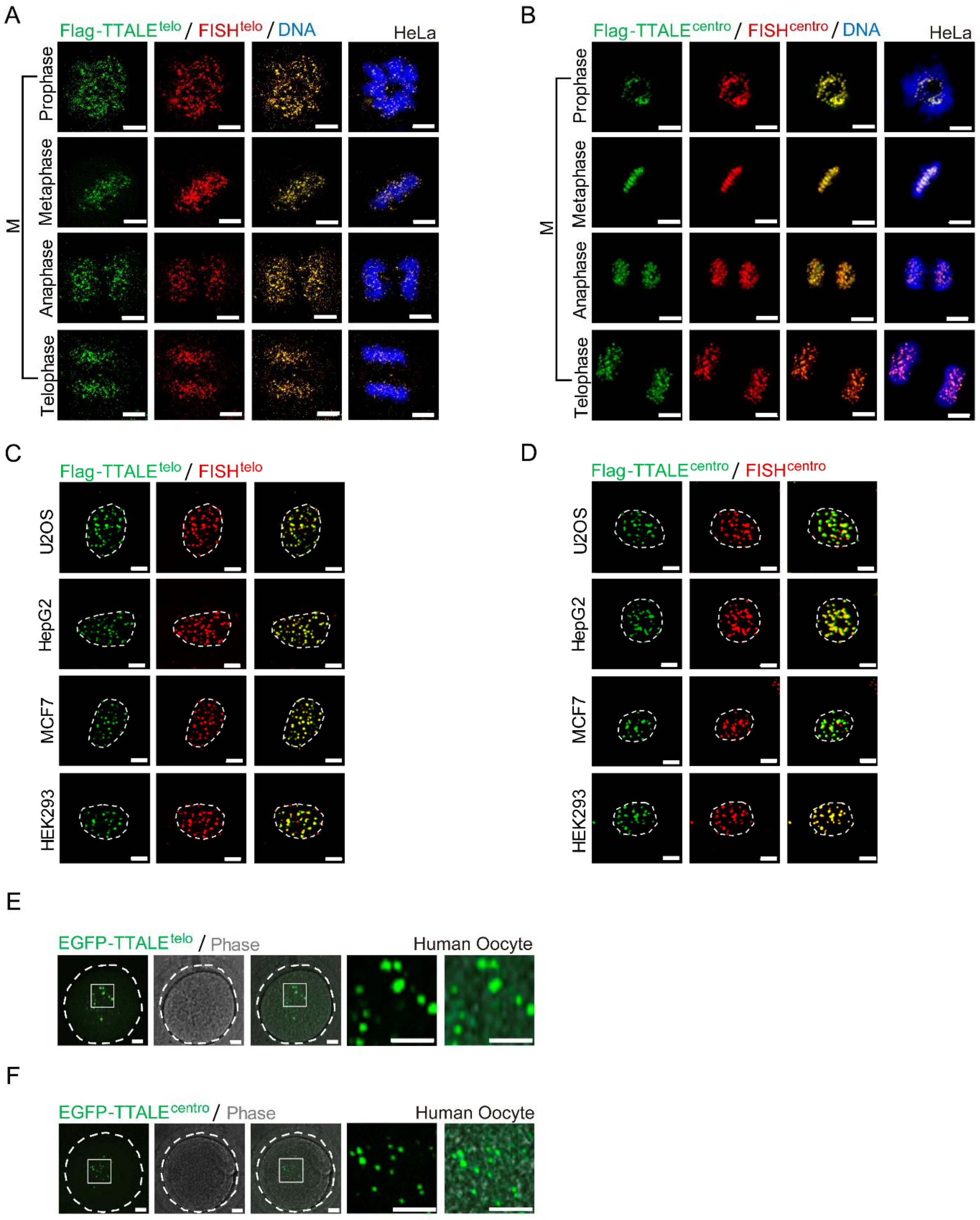

Supplementary information, Figure S4 TTALE-based imaging of telomeres and centromeres during mitosis and in different human cell types. **(A-B)** Co-localization analysis of telomeric FISH (red) and Flag-TTALE^{telo} (green) (A) or centromeric FISH (red) and Flag-TTALE^{centro} (green) (B) in HeLa cells at different mitotic stages. Scale bars, 5 μm. **(C-D)** Co-localization analysis of telomeric FISH (red) and Flag-TTALE^{telo} (green) (C) or centromeric FISH (red) and Flag-TTALE^{centro} (green) (D) in U2OS, HepG2, MCF7, and HEK293 cells. Scale bars, 5 μm. **(E-F)** TTALE-mediated imaging of telomeres (E) and centromeres (F) in live human oocytes. Dashed lines indicate cellular boundaries. Scale bars, 10 μm.
