# Supplementary material for: Visualization of aging-associated chromatin alterations with an engineered TALE system
Source: Cell Res. 2017 Jan 31;27(4):483–504. doi: 10.1038/cr.2017.18 (PMC5385610; doi:10.1038/cr.2017.18)
Supplement: Supplementary information, Figure S5 — Characterization of human pluripotent stem cells and their differentiated derivatives. [file cr201718x5.pdf]

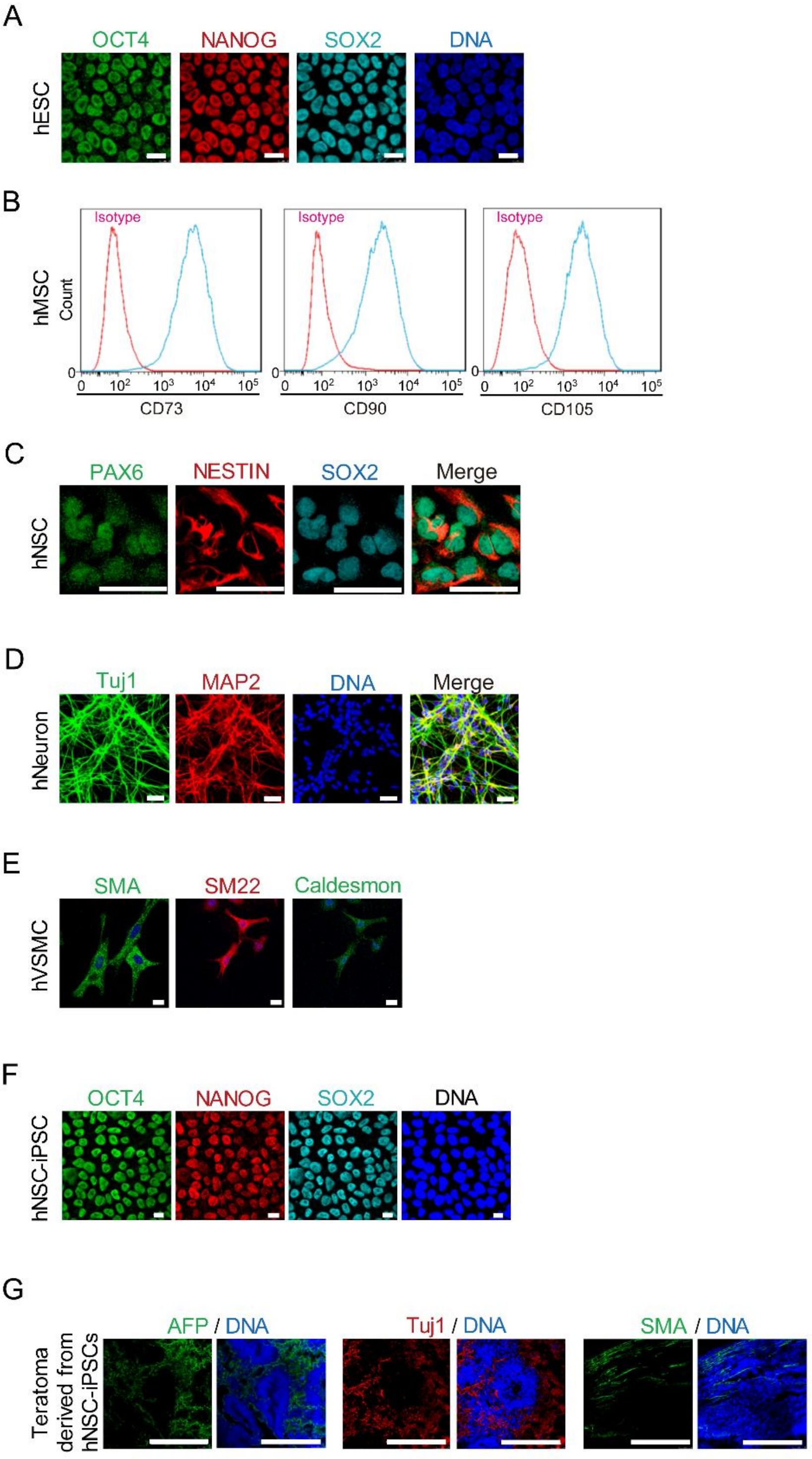

Supplementary information, Figure S5 Characterization of human pluripotent stem cells and their differentiated derivatives. **(A)** Immunofluorescence images showing expression of the pluripotency markers OCT4, NANOG, and SOX2 in hESCs. Nuclei were counterstained with Hoechst 33342. Scale bars, 25 μm. **(B)** Flow cytometry analysis of the surface markers CD73, CD90, and CD105 in hMSCs. Red, isotype control; cyan, antibody-stained cells. **(C-G)** Immunofluorescence images showing expression of PAX6, NESTIN, and SOX2 in hNSCs (C); Tuj1 and MAP2 in human postmitotic neurons (D); SMA, SM22, and caldesmon in hVSMCs (E); pluripotency markers OCT4, NANOG, and SOX2 in iPSCs reprogrammed from hNSCs (nuclei were counterstained with DAPI) (F) ; and endoderm marker AFP (green), exoderm marker Tuj1 (red), and mesoderm marker SMA (green) in teratomas derived from hNSC-iPSCs (G). Scale bars, 25 μm (C-F) or 10 μm (G).
