# Supplementary material for: Visualization of aging-associated chromatin alterations with an engineered TALE system
Source: Cell Res. 2017 Jan 31;27(4):483–504. doi: 10.1038/cr.2017.18 (PMC5385610; doi:10.1038/cr.2017.18)
Supplement: Supplementary information, Figure S6 — TTALE-based imaging of NOR-rDNAs and MUC4. [file cr201718x6.pdf]

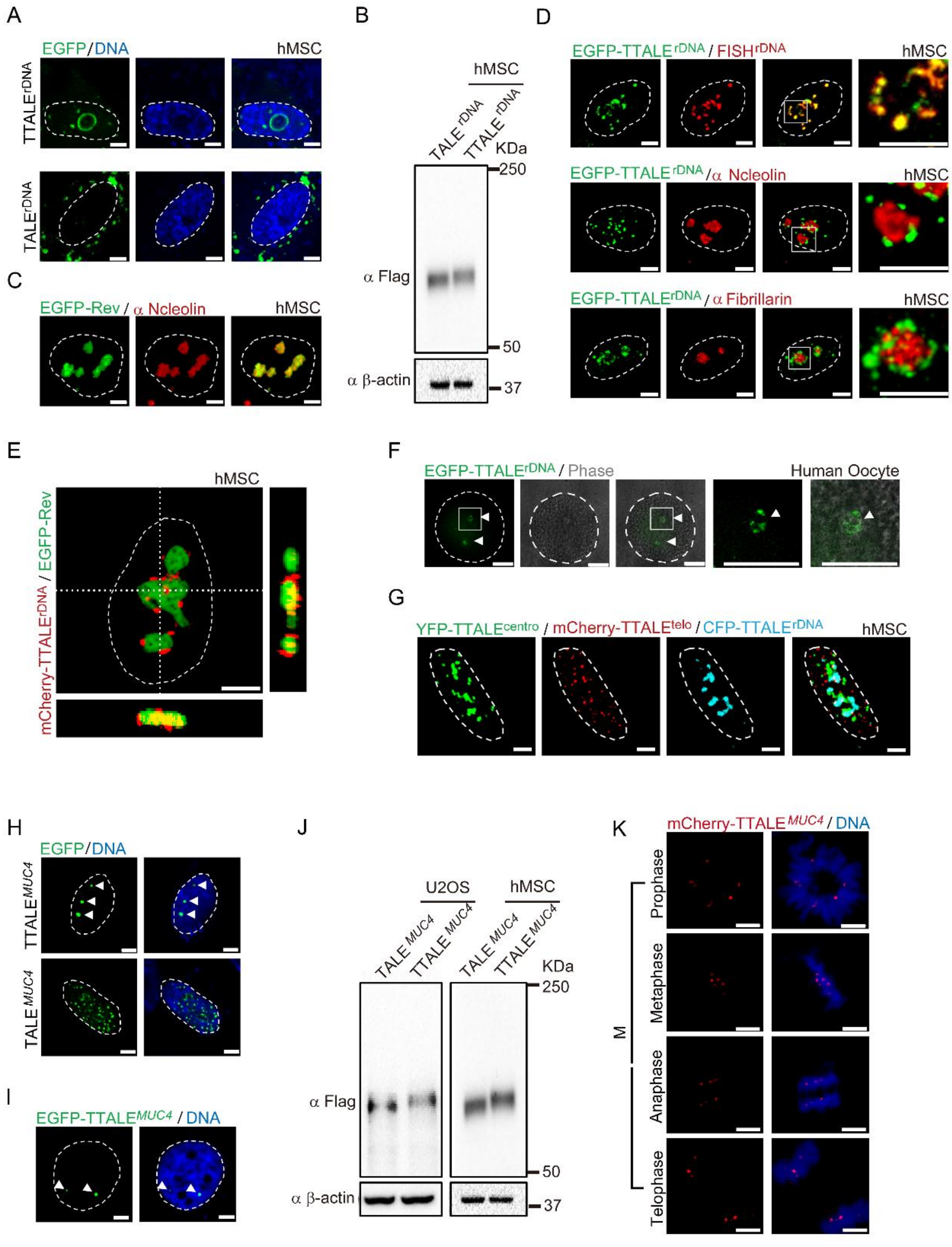

Supplementary information, Figure S6 TTALE-based imaging of NOR-rDNAs and *MUC4*. **(A)** Localization of unmodified EGFP-TALE^{rDNA} (bottom) and EGFP-TTALE^{rDNA} (top) in hMSCs. Dashed lines indicate the nuclear boundary. Scale bars, 5 μm. **(B)** Western blots demonstrating correct expression of TALE^{rDNA} and TTALE^{rDNA} in hMSCs. β-actin was used as a loading control. **(C)** Co-localization of EGFP-Rev (green) and nucleolin (red). Dashed lines indicate the nuclear boundary. Scale bars, 5 μm. **(D)** Co-localization of EGFP-TTALE^{rDNA} (green) and rDNA FISH (top), nucleolin (middle), or fibrillarin (bottom) (red) in hMSCs. Dashed lines indicate the nuclear boundary. Scale bars, 5 μm. **(E)** Live cell imaging of mCherry-TTALE^{rDNA} (red) and EGFP-Rev (green) in hMSCs. Slice view of stacks along the two dotted lines are shown respectively on the right or bottom. Scale bars, 5 μm. **(F)** TTALE^{rDNA}-mediated visualization of NOR-rDNA in live human oocytes. Dashed lines indicate the cellular boundary. Arrowheads indicate NOR-rDNA loci. Scale bars, 33 μm. **(G)** Simultaneous visualization of telomeres, centromeres, and rDNAs in a single live cell using mCherry-TTALE^{telo}, YFP-TTALE^{centro}, and CFP-TTALE^{rDNA}. Dashed lines indicate the nuclear boundary. Scale bars, 5 μm. **(H-I)** Unmodified TALE^{MUC4} (bottom) and TTALE^{MUC4} (top) in HeLa cells (H) and hMSCs (I). Dashed lines indicate the nuclear boundary; arrowheads indicate *MUC4* loci. Scale bars, 5 μm. **(J)** Western blots showing correct expression of TALE^{MUC4} and TTALE^{MUC4} in U2OS cells or wild type hMSCs. β-actin was used as a loading control. **(K)** Visualization of *MUC4* locus at different stages of mitosis by mCherry-TTALE^{MUC4} (red) in HeLa cells. Hoechst was used to label DNA (blue). Scale bars, 5 μm.
