# Supplementary material for: Visualization of aging-associated chromatin alterations with an engineered TALE system
Source: Cell Res. 2017 Jan 31;27(4):483–504. doi: 10.1038/cr.2017.18 (PMC5385610; doi:10.1038/cr.2017.18)
Supplement: Supplementary information, Figure S7 — TTALE-mediated visualization of telomeric and centromeric changes in human stem cell aging models. [file cr201718x7.pdf]

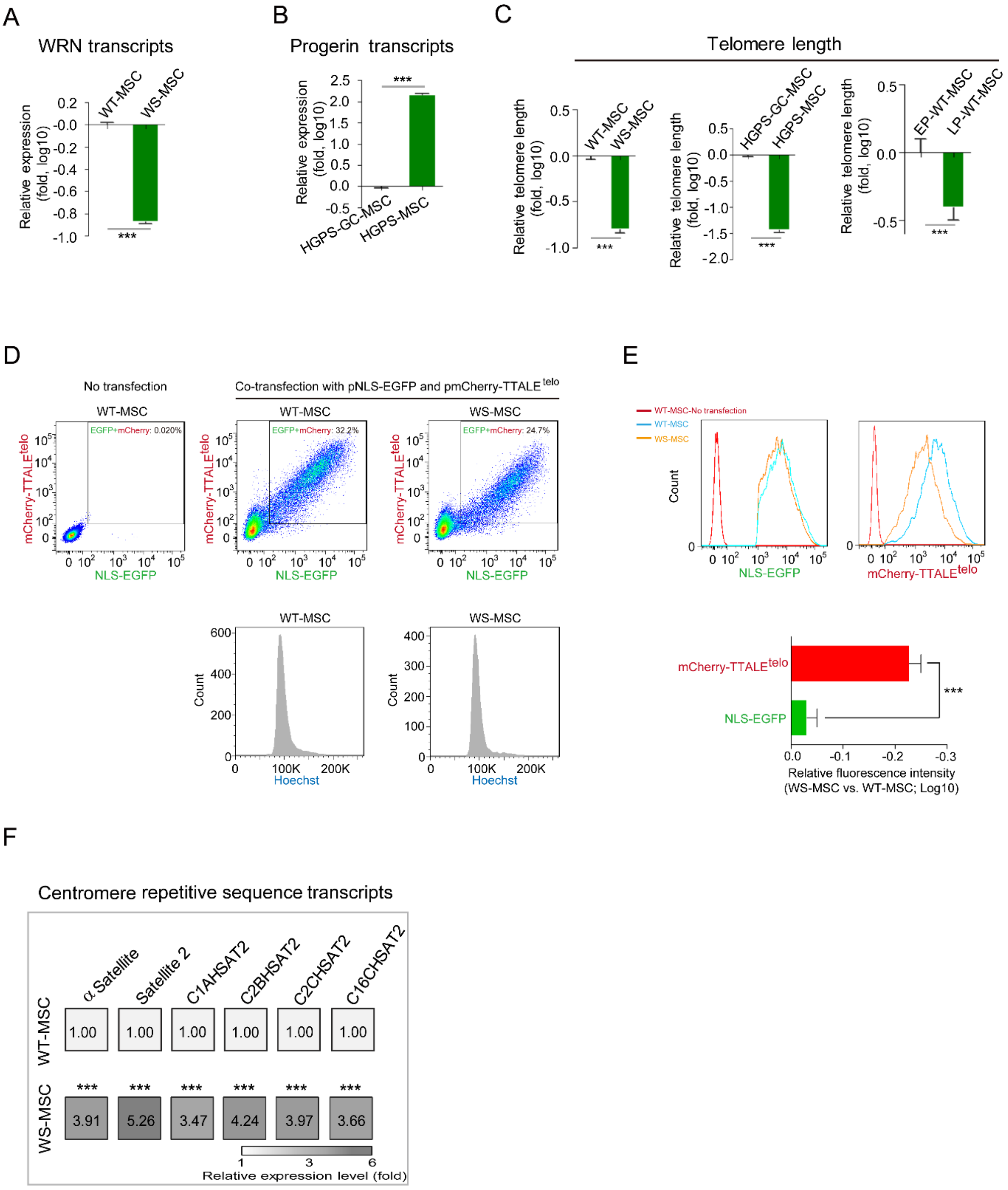


**Supplementary information, Figure S7** TTALE-mediated visualization of telomeric and centromeric changes in human stem cell aging models. **(A-B)** RT-qPCR analysis of expression of WRN (A) and progerin (B) in the indicated cell types. Values were normalized to 18S rRNA. Data were presented as mean ± SEM; n = 3; ***p < 0.001. **(C)** Telomere length in hMSCs detected by qPCR in the indicated cell types. Values were normalized to 36B4. Data were presented as mean ± SEM; n = 3; ***p < 0.001. **(D)** FACS analysis of hMSCs co-transfected with mCherry-TTALE$^{telo}$ and NLS-EGFP. Top: the boxed regions show dual-labeled cells in WT-MSCs without tranfection, WT-MSCs transfected with mCherry-TTALE$^{telo}$ and NLS-EGFP, and WS-MSCs transfected with mCherry-TTALE$^{telo}$ and NLS-EGFP. Bottom: histograms indicating that Hoechst staining signals were comparable between WT-MSCs and WS-MSCs. **(E)** FACS analysis of hMSCs co-transfected with mCherry-TTALE$^{telo}$ and NLS-EGFP (See D) showed lower mCherry-TTALE$^{telo}$ rather than NLS-EGFP (transfection internal control) florescence intensity in WS-MSCs relative to WT-MSCs. The bottom histogram showed a decrease in average fluorescence intensity of mCherry-TTALE$^{telo}$ in WS-MSCs compared to WT-MSCs. Data were presented as mean ± SEM; n = 4; ***p<0.001. **(F)** RT-qPCR analysis of centromeric repetitive sequence transcripts in WS-MSCs and WT-MSCs. Values were normalized to GAPDH. Data were presented as mean ± SEM; n = 3; ***p < 0.001.
