# Supplementary material for: Visualization of aging-associated chromatin alterations with an engineered TALE system
Source: Cell Res. 2017 Jan 31;27(4):483–504. doi: 10.1038/cr.2017.18 (PMC5385610; doi:10.1038/cr.2017.18)
Supplement: Supplementary information, Figure S8 — TTALE-mediated visualization of telomeres and centromeres in WRN-deficient ESCs and NSCs. [file cr201718x8.pdf]

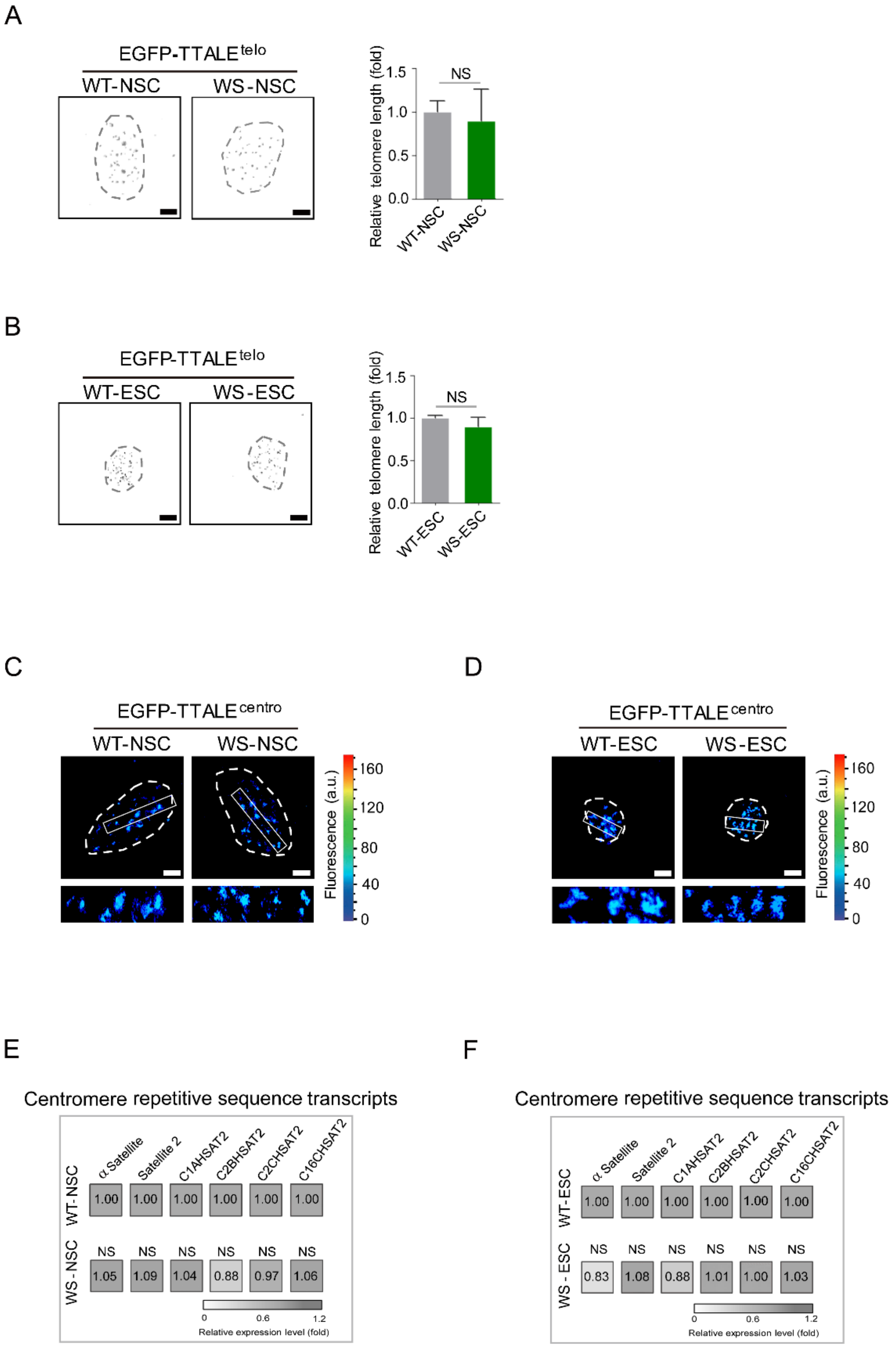

Supplementary information, Figure S8 TTALE-mediated visualization of telomeres and centromeres in WRN-deficient ESCs and NSCs. **(A-B)** Left: EGFP-TTALE^{telo}-based SIM imaging showing comparable telomere lengths between WS-NSCs and WT-NSCs (A) and between WS-ESCs and WT-ESCs (B). Dashed lines indicate the nuclear boundary. Scale bars, 5 μm. Right: Telomere length detected by qPCR. Values were normalized to 36B4. Data were presented as mean ± SEM; n = 3; NS, not significant. **(C-D)** Top: SIM images showing centromeres labeled by EGFP-TTALE^{centro} in NSCs (C) and ESCs (D). Bottom: Magnified images of boxed regions from top panels. Dashed lines indicate the nuclear boundary. Scale bars, 5 μm. **(E-F)** RT-qPCR analysis of centromeric repetitive sequence transcripts in NSCs (E) and ESCs (F). Values were normalized to GAPDH. Data were presented as mean ± SEM; n = 3; NS, not significant.
