# Supplementary material for: Visualization of aging-associated chromatin alterations with an engineered TALE system
Source: Cell Res. 2017 Jan 31;27(4):483–504. doi: 10.1038/cr.2017.18 (PMC5385610; doi:10.1038/cr.2017.18)
Supplement: Supplementary information, Figure S9 — TTALE-based imaging of attrition of NOR-rDNAs in senescenth MSCs. [file cr201718x9.pdf]

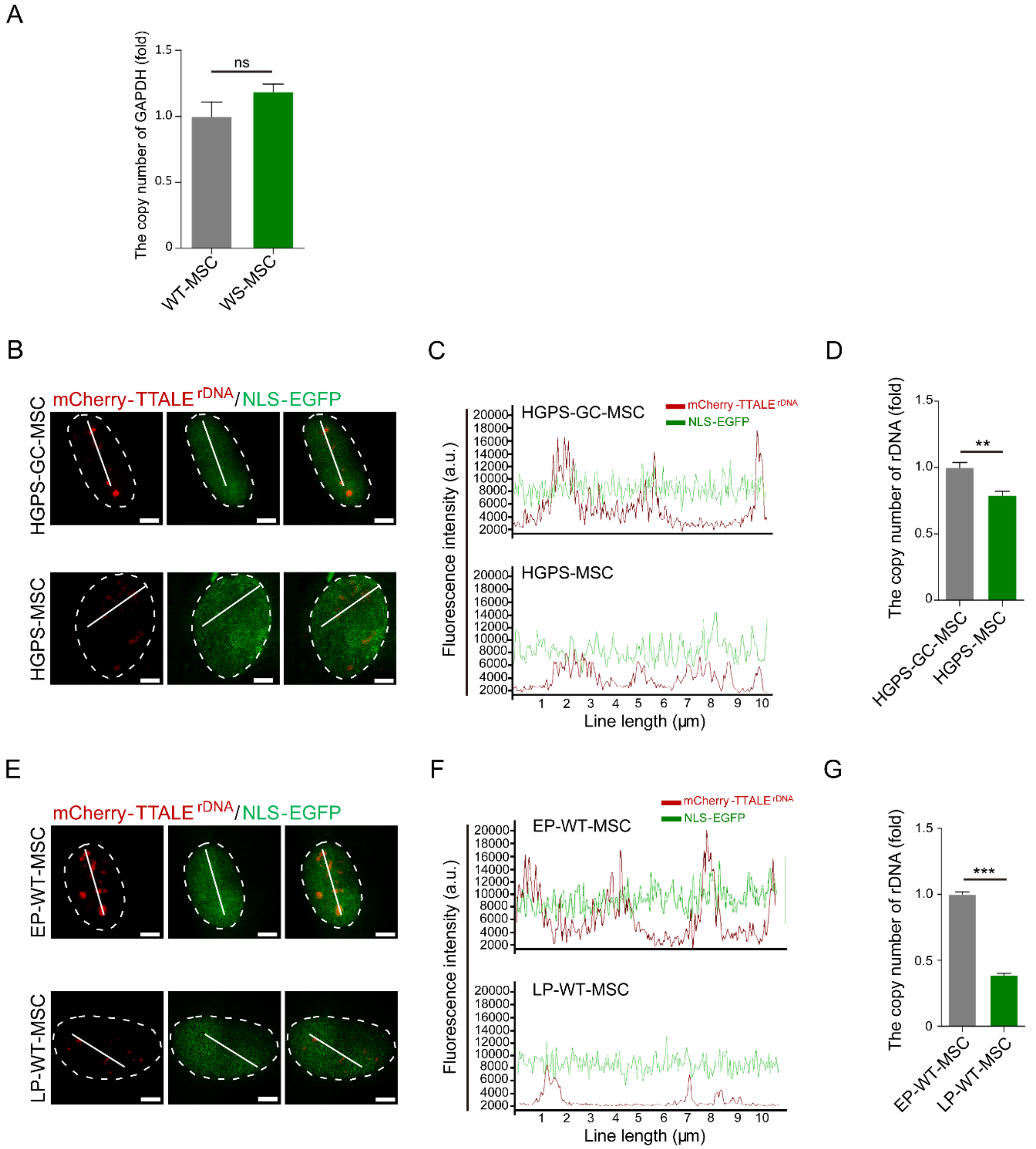

Supplementary information, Figure S9 TTALE-based imaging of attrition of NOR-rDNAs in senescent hMSCs. **(A)** Quantitative PCR analysis showing no significant difference of *GAPDH* gene copy number in WS-MSCs relative to WT-MSCs. Data were presented as mean ± SEM; n = 3; NS, not significant. **(B, E)** SIM images showing diminished mCherry-TTALE^{rDNA} fluorescence in HGPS-MSCs (passage 8) compared to HGPS-GC-MSCs (passage 8) (B) and LP-WT-MSCs (passage 12) compared to EP-WT-MSCs (passage 6) (E) . NLS-EGFP was co-transfected as an internal control. Dashed lines indicate the nuclear boundary. Scale bars, 5 μm. **(C, F)** Intensity profiles across TTALE^{rDNA}-labeled rDNAs in HGPS-GC-MSCs and HGPS-MSCs (C) and EP-WT-MSCs and LP-WT-MSCs (F) as indicated by solid lines in Figure S9B and S9E, respectively. Solid lines represent 10 μm in length. **(D, G)** qPCR analysis indicating a reduction in rDNA copy number in HGPS-MSCs relative to WT control cells (D) and LP-WT-MSCs (passage 12) compared to EP-WT-MSCs (passage 6) (G) . Data were presented as mean ± SEM; n = 3; **p < 0.01; ***p < 0.001.
