# Supplementary material for: Visualization of aging-associated chromatin alterations with an engineered TALE system
Source: Cell Res. 2017 Jan 31;27(4):483–504. doi: 10.1038/cr.2017.18 (PMC5385610; doi:10.1038/cr.2017.18)
Supplement: Supplementary information, Figure S10 — TTALE-mediated visualization of aging-associated DDRs at telomeric and centromeric loci. [file cr201718x10.pdf]

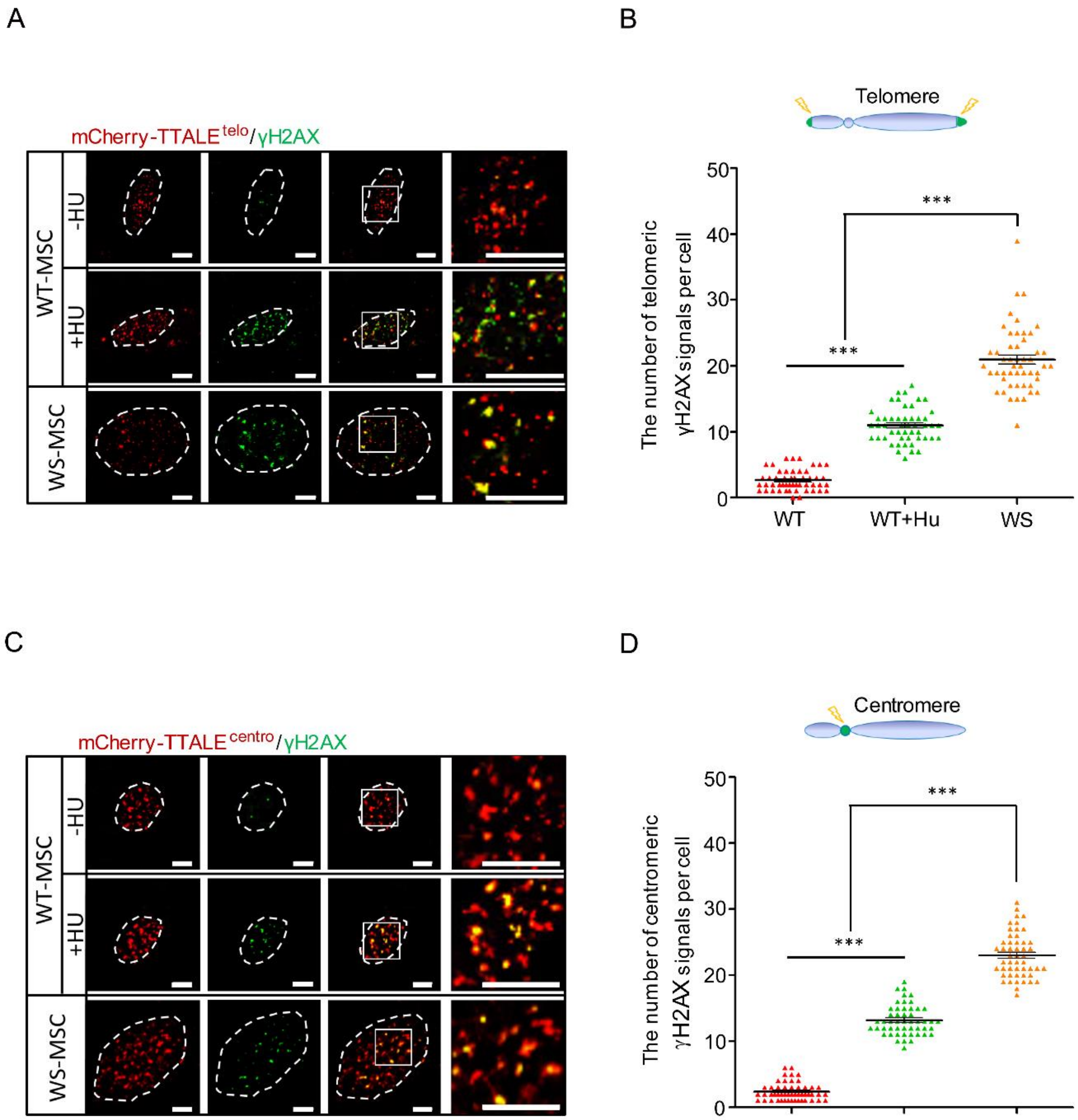

Supplementary information, Figure S10 TTALE-mediated visualization of aging-associated DDRs at telomeric and centromeric loci. **(A, C)** Immunofluorescence analysis of γ-H2AX (green) expression in mCherry-TTALE^{telo}-transfected (red) (A) and mCherry-TTALE^{centro}-transfected (red) (C) WT and WS hMSCs. HU treatment (10 nM, 2 hours) was used as a positive control to induce γ-H2AX. Dashed lines indicate the nuclear boundary. Scale bars, 5 μm. **(B, D)** Number of γ-H2AX-positive telomeres (B) or centromeres (D) per cell in WS hMSCs, untreated WT hMSCs, and WT hMSCs treated with HU. n = 50 nuclei per group; ***$p < 0.001$.
