# Supplementary material for: Visualization of aging-associated chromatin alterations with an engineered TALE system
Source: Cell Res. 2017 Jan 31;27(4):483–504. doi: 10.1038/cr.2017.18 (PMC5385610; doi:10.1038/cr.2017.18)
Supplement: Supplementary information, Figure S11 — Lentiviral TTALEtelo-mediated in vivo imaging of telomeres in telomerase-deficient mice. [file cr201718x11.pdf]

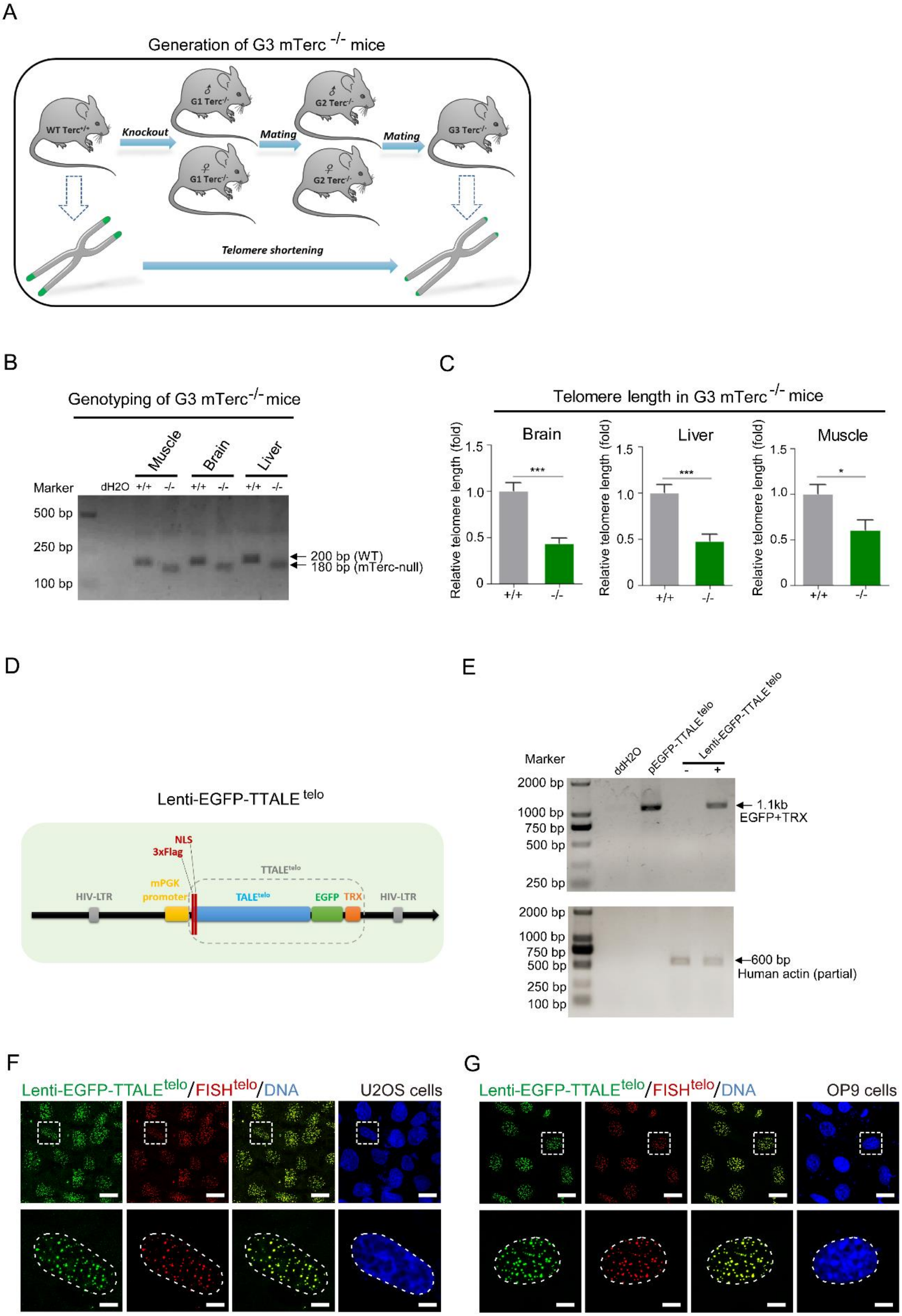

Supplementary information, Figure S11 Lentiviral TTALE^{telo}-mediated *in vivo* imaging of telomeres in telomerase-deficient mice. **(A)** Flow chart of generation of G3 *mTerc*$^{-/-}$ mice with shortened telomeres. **(B)** Genotyping of G3 *mTerc*$^{-/-}$ mice in different tissues including muscle, brain, and liver. **(C)** qPCR detection of telomere length in brain (left), liver (middle), and muscle (right) of WT *mTerc*$^{+/+}$ and G3 *mTerc*$^{-/-}$ mice. Values were normalized to *Mapk1*. Data were presented as mean ± SEM; n = 4; $^{*}p < 0.05$; $^{***}p < 0.001$. **(D)** Schematic illustration of the EGFP-TTALE cassette in a lentiviral vector for telomere labeling. **(E)** PCR verifying the integration of EGFP-TTALE^{telo} cassette in the genomic DNA extracted from U2OS cells transduced with or without lentiviral EGFP-TTALE^{telo} vector. Purified plasmid EGFP-TTALE^{telo} was used as a positive PCR template. **(F-G)** Co-localization of EGFP-TTALE^{telo} (green) and FISH^{telo} (red) signals in human U2OS cells (F) and mouse OP9 cells (G) transduced with lentiviral EGFP-TTALE^{telo} vector. Scale bars：30 μm (top); 5 μm (bottom).
