# Supplementary material for: Visualization of aging-associated chromatin alterations with an engineered TALE system
Source: Cell Res. 2017 Jan 31;27(4):483–504. doi: 10.1038/cr.2017.18 (PMC5385610; doi:10.1038/cr.2017.18)
Supplement: Supplementary information, Data S1 — DNA sequencing of Lenti-EGFP-TTALEtelo construct demonstrating no recombination. [file cr201718x15.pdf]

Supplementary information, Data S1 DNA sequencing of Lenti-EGFP-TTALEtelo construct demonstrating no recombination.

caggtggcacttttcggggaaatgtgcgcggaacccctatttgtttatttttctaaatacattcaaatatgtatccgctcatgagacaataaccc
tgataaatgcttcaataatattgaaaaaggaagagtatgagtattcaacatttccgtgtcgcccttattcccttttttgcggcattttgccttcctg
tttttgctcacccagaaacgctggtgaaagtaaaagatgctgaagatcagttgggtgcacgagtgggttacatcgaactggatctcaacagc
ggtaagatccttgagagttttcgccccgaagaacgttttccaatgatgagcacttttaaagttctgctatgtggcgcggtattatcccgtattgac
gccgggcaagagcaactcggtcgccgcatacactattctcagaatgacttggttgagtactcaccagtcacagaaaagcatcttacggatgg
catgacagtaagagaattatgcagtgctgccataaccatgagtgataacactgcggccaacttacttctgacaacgatcggaggaccgaag
gagctaaccgcttttttgcacaacatgggggatcatgtaactcgccttgatcgttgggaaccggagctgaatgaagccataccaaacgacga
gcgtgacaccacgatgcctgtagcaatggcaacaacgttgcgcaaactattaactggcgaactacttactctagcttcccggcaacaattaat
agactggatggaggcggataaagttgcaggaccacttctgcgctcggcccttccggctggctggtttattgctgataaatctggagccggtga
gcgtgggtctcgcggtatcattgcagcactggggccagatggtaagccctcccgtatcgtagttatctacacgacggggagtcaggcaactat
ggatgaacgaaatagacagatcgctgagataggtgcctcactgattaagcattggtaactgtcagaccaagtttactcatatatactttagatt
gatttaaaacttcatttttaatttaaaaggatctaggtgaagatcctttttgataatctcatgaccaaaatcccttaacgtgagttttcgttccact
gagcgtcagaccccgtagaaaagatcaaaggatcttcttgagatcctttttttctgcgcgtaatctgctgcttgcaaacaaaaaaaccaccgct
accagcggtggtttgtttgccggatcaagagctaccaactctttttccgaaggtaactggcttcagcagagcgcagataccaaatactgtcctt
ctagtgtagccgtagttaggccaccacttcaagaactctgtagcaccgcctacatacctcgctctgctaatcctgttaccagtggctgctgcca
gtggcgataagtcgtgtcttaccgggttggactcaagacgatagttaccggataaggcgcagcggtcgggctgaacggggggttcgtgcac
acagcccagcttggagcgaacgacctacaccgaactgagatacctacagcgtgagctatgagaaagcgccacgcttcccgaagggagaaa
ggcggacaggtatccggtaagcggcagggtcggaacaggagagcgcacgagggagcttccagggggaaacgcctggtatctttatagtcc
tgtcgggtttcgccacctctgacttgagcgtcgatttttgtgatgctcgtcaggggggcggagcctatggaaaaacgccagcaacgcggccttt
ttacggttcctggccttttgctggccttttgctcacatgttctttcctgcgttatcccctgattctgtggataaccgtattaccgcctttgagtgagct
gataccgctcgccgcagccgaacgaccgagcgcagcgagtcagtgagcgaggaagcggaagagcgcccaatacgcaaaccgcctctccc
cgcgcgttggccgattcattaatgcagctggcacgacaggtttcccgactggaaagcgggcagtgagcgcaacgcaattaatgtgagttagc
tcactcattaggcaccccaggctttacactttatgcttccggctcgtatgttgtgtggaattgtgagcggataacaatttcacacaggaaacag
ctatgaccatgattacgccaagcgcgcaattaaccctcactaaagggaacaaaagctggagctgcaagcttaatgtagtcttatgcaatact
cttgtagtcttgcaacatggtaacgatgagttagcaacatgccttacaaggagagaaaaagcaccgtgcatgccgattggtggaagtaaggt
ggtacgatcgtgccttattaggaaggcaacagacgggtctgacatggattggacgaaccactgaattgccgcattgcagagatattgtattta
agtgcctagctcgatacaataaacgggtctctctggttagaccagatctgagcctgggagctctctggctaactagggaacccactgcttaag
cctcaataaagcttgccttgagtgcttcaagtagtgtgtgcccgtctgttgtgtgactctggtaactagagatccctcagacccttttagtcagtg
tggaaaatctctagcagtggcgcccgaacagggacctgaaagcgaaagggaaaccagagctctctcgacgcaggactcggcttgctgaag
cgcgcacggcaagaggcgaggggcggcgactggtgagtacgccaaaaattttgactagcggaggctagaaggagagagatgggtgcgag
agcgtcagtattaagcgggggagaattagatcgcgatgggaaaaaattcggttaaggccagggggaaagaaaaaatataaattaaaacat
atagtatgggcaagcagggagctagaacgattcgcagttaatcctggcctgttagaaacatcagaaggctgtagacaaatactgggacagc
tacaaccatcccttcagacaggatcagaagaacttagatcattatataatacagtagcaaccctctattgtgtgcatcaaaggatagagataa
aagacaccaaggaagctttagacaagatagaggaagagcaaaacaaaagtaagaccaccgcacagcaagcggccgctgatcttcagacc
tggaggaggagatatgagggacaattggagaagtgaattatataaatataaagtagtaaaaattgaaccattaggagtagcacccaccaa
ggcaaagagaagagtggtgcagagagaaaaaagagcagtgggaataggagctttgttccttgggttcttgggagcagcaggaagcactat
gggcgcagcctcaatgacgctgacggtacaggccagacaattattgtctggtatagtgcagcagcagaacaatttgctgagggctattgagg
cgcaacagcatctgttgcaactcacagtctggggcatcaagcagctccaggcaagaatcctggctgtggaaagatacctaaaggatcaaca
gctcctggggatttggggttgctctggaaaactcatttgcaccactgctgtgccttggaatgctagttggagtaataaatctctggaacagatt
ggaatcacacgacctggatggagtgggacagagaaattaacaattacacaagcttaatacactccttaattgaagaatcgcaaaaccagca
agaaaagaatgaacaagaattattggaattagataaatgggcaagtttgtggaattggtttaacataacaaattggctgtggtatataaaatt
attcataatgatagtaggaggcttggtaggtttaagaatagtttttgctgtactttctatagtgaatagagttaggcagggatattcaccattatc

gtttcagacccacctcccaaccccgaggggacccgacaggcccgaaggaatagaagaagaaggtggagagagagacagagacagatcc
attcgattagtgaacggatctcgacggttaacttttaaaagaaaaggggggattggggggtacagtgcaggggaaagaatagtagacataa
tagcaacagacatacaaactaaagaattacaaaaacaaattacaaaaattcaaaattttatcgatggtacctaccgggtaggggaggcgct
tttcccaaggcagtctggagcatgcgctttagcagccccgctgggcacttggcgctacacaagtggcctctggctcgcacacattccacatcc
accggtaggcgccaaccggctccgttctttggtggccccttcgcgccaccttctactcctcccctagtcaggaagttcccccccgccccgcagc
tcgcgtcgtgcaggacgtgacaaatggaagtagcacgtctcactagtctcgtgcagatggacagcaccgctgagcaatggaagcdggtagg
cctttggggcagcggccaatagcagctttgctccttcgctttctgggctcagaggctgggaaggggtgggtccgggggcgggctcaggggcg
ggctcaggggcggggcgggcgcccgaagtcctccggaggcccggcattctgcacgcttcaaaagcgcacgtctgccgcgctgttctcctctt
cctcatctccgggcctttcgacctgcactctagaggatccctcgagaccggtcgccaccgagctctctggctaactagagaacccactgcttac
tggcttatcgaaattaatacgactcactatagggccaccatggactataaggaccacgacggagactacaaggatcatgatattgattac
aaagacgatgacgataagatggccccaaagaagaagcggaaggtcggtatccacggagtcccagcagccGTAGATTTGAGAACT
TTGGGATATTCACAGCAGCAGCAGGAAAAGATCAAGCCCAAAGTGAGGTCGACAGTCGCGCAGCATCACG
AAGCGCTGGTGGGTCATGGGTTTACACATGCCCACATCGTAGCCTTGTCGCAGCACCCTGCAGCCCTTGGC
ACGGTCGCCGTCAAGTACCAGGACATGATTGCGGCGTTGCCGGAAGCCACACATGAGGCGATCGTCGGTG
TGGGGAAACAGTGGAGCGGAGCCCGAGCGCTTGAGGCCCTGTTGACGGTCGCGGGAGAGCTGAGAGGG
CCTCCCCTTCAGCTGGACACGGGCCAGTTGCTGAAGATCGCGAAGCGGGGAGGAGTCACGGCGGTCGAG
GCGGTGCACGCGTGGCGCAATGCGCTCACGGGAGCACCCCTCAACCTGACCCCAGAGCAGGTCGTGGCAA
TTGCGAGCAACATCGGGGGAAAGCAGGCACTCGAAACCGTCCAGAGGTTGCTGCCTGTGCTGTGCCAAGC
GCACGGACTTACGCCAGAGCAGGTCGTGGCAATTGCGAGCAACATCGGGGGAAAGCAGGCACTCGAAAC
CGTCCAGAGGTTGCTGCCTGTGCTGTGCCAAGCGCACGGACTAACCCCAGAGCAGGTCGTGGCAATTGCG
AGCCATGACGGGGGAAAGCAGGCACTCGAAACCGTCCAGAGGTTGCTGCCTGTGCTGTGCCAAGCGCAC
GGGTTGACCCCAGAGCAGGTCGTGGCAATTGCGAGCCATGACGGGGGAAAGCAGGCACTCGAAACCGTC
CAGAGGTTGCTGCCTGTGCTGTGCCAAGCGCACGGCCTGACCCCAGAGCAGGTCGTGGCAATTGCGAGCC
ATGACGGGGGAAAGCAGGCACTCGAAACCGTCCAGAGGTTGCTGCCTGTGCTGTGCCAAGCGCACGGAC
TGACACCAGAGCAGGTCGTGGCAATTGCGAGCAACGGAGGGGGAAAGCAGGCACTCGAAACCGTCCAGA
GGTTGCTGCCTGTGCTGTGCCAAGCGCACGGACTTACACCCGAACAAGTCGTGGCAATTGCGAGCAACATC
GGGGGAAAGCAGGCACTCGAAACCGTCCAGAGGTTGCTGCCTGTGCTGTGCCAAGCGCACGGACTTACG
CCAGAGCAGGTCGTGGCAATTGCGAGCAACATCGGGGGAAAGCAGGCACTCGAAACCGTCCAGAGGTTG
CTGCCTGTGCTGTGCCAAGCGCACGGACTAACCCCAGAGCAGGTCGTGGCAATTGCGAGCCATGACGGGG
GAAAGCAGGCACTCGAAACCGTCCAGAGGTTGCTGCCTGTGCTGTGCCAAGCGCACGGGTTGACCCCAGA
GCAGGTCGTGGCAATTGCGAGCCATGACGGGGGAAAGCAGGCACTCGAAACCGTCCAGAGGTTGCTGCC
TGTGCTGTGCCAAGCGCACGGCCTGACCCCAGAGCAGGTCGTGGCAATTGCGAGCCATGACGGGGGAAA
GCAGGCACTCGAACCGTCCAGAGGTTGCTGCCTGTGCTGTGCCAAGCGCACGGACTGACACCAGAGCAGG
TCGTGGCAATTGCGAGCAACGGAGGGGGAAAGCAGGCACTCGAAACCGTCCAGAGGTTGCTGCCTGTGC
TGTGCCAAGCGCACGGCCTCACCCCAGAGCAGGTCGTGGCAATTGCGAGCAACATCGGGGGAAAGCAGG
CACTCGAAACCGTCCAGAGGTTGCTGCCTGTGCTGTGCCAAGCGCACGGACTTACGCCAGAGCAGGTCGT
GGCAATTGCGAGCAACATCGGGGGAAAGCAGGCACTCGAAACCGTCCAGAGGTTGCTGCCTGTGCTGTGC
CAAGCGCACGGACTAACCCCAGAGCAGGTCGTGGCAATTGCGAGCCATGACGGGGGAAAGCAGGCACTC
GAAACCGTCCAGAGGTTGCTGCCTGTGCTGTGCCAAGCGCACGGGTTGACCCCAGAGCAGGTCGTGGCAA
TTGCGAGCCATGACGGGGGAAAGCAGGCACTCGAAACCGTCCAGAGGTTGCTGCCTGTGCTGTGCCAAGC
GCACGGCCTGACCCCAGAGCAGGTCGTGGCAATTGCGAGCCATGACGGGGGAAAGCAGGCACTCGAAAC
CGTCCAGAGGTTGCTGCCTGTGCTGTGCCAAGCGCACGGACTGACACCAGAGCAGGTCGTGGCAATTGCG
AGCAACGGAGGGGGAAAGCAGGCACTCGAAACCGTCCAGAGGTTGCTGCCTGTGCTGTGCCAAGCGCAC
GGACTCACGCCTGAGCAGGTAGTGGCTATTGCATCCAACATCGGGGGCAGACCCGCACTGGAGTCAATCGT

mPGK promoter

3xFlag

NLS

TALE

RVD

GGCCCAGCTTTCGAGGCCGGACCCCGCGCTGGCCGCACTCACTAATGATCATCTTGTAGCGCTGGCCTGCC
TCGGCGGACGACCCGCCTTGGATGCGGTGAAGAAGGGGCTCCCGCACGCGCCTGCATTGATTAAGCGGAC
CAACAGAAGGATTCCCGAGAGGACATCACATCGAGTGGCAAGTTATCAGGGAatggtgagcaagggcgaggagct
gttcaccggggtggtgcccatcctggtcgagctggacggcgacgtaaacggccacaagttcagcgtgtccggcgagggcgagggcgatgcc EGFP
acctacggcaagctgaccctgaagttcatctgcaccaccggcaagctgcccgtgccctggcccaccctcgtgaccaccctgacctacggcgt
gcagtgcttcagccgctaccccgaccacatgaagcagcacgacttcttcaagtccgccatgcccgaaggctacgtccaggagcgcaccatct
tcttcaaggacgacggcaactacaagacccgcgccgaggtgaagttcgagggcgacaccctggtgaaccgcatcgagctgaagggcatcg
acttcaaggaggacggcaacatcctggggcacaagctggagtacaactacaacagccacaacgtctatatcatggccgacaagcagaaga
acggcatcaaggtgaacttcaagatccgccacaacatcgaggacggcagcgtgcagctcgccgaccactaccagcagaacacccccatcg
gcgacggccccgtgctgctgcccgacaaccactacctgagcacccagtccgccctgagcaaagaccccaacgagaagcgcgatcacatgg
tcctgctggagttcgtgaccgccgccgggatcactctcggcatggacgagctgtacaagtccggactcagatctcgagctcaagcttcgaatt TRX
ccatggtgaagcagatcgagagcaagactgcttttcaggaagccttggacgctgcaggtgataaacttgtagtagttgacttctcagccacgt
ggtgtgggccttgcaaaatgatcaagcctttctttcattccctctctgaaaagtattccaacgtgatattccttgaagtagatgtggatgactgtc
aggatgttgcttcagagtgtgaagtcaaatgcatgccaacattccagtttttaagaagggacaaaaggtgggtgaattttctggagccaata
aggaaaagcttgaagccaccattaatgaattagtctaactcgagagcggccgcgacgcgtgtcgacaatcaacctctggattacaaaatttg
tgaaagattgactggtattcttaactatgttgctccttttacgctatgtggatacgctgctttaatgcctttgtatcatgctattgcttcccgtatgg
ctttcattttctcctccttgtataaatcctggttgctgtctctttatgaggagttgtggcccgttgtcaggcaacgtggcgtggtgtgcactgtgttt
gctgacgcaacccccactggttggggcattgccaccacctgtcagctcctttccgggactttcgctttccccctccctattgccacggcggaact
catcgccgcctgccttgcccgctgctggacaggggctcggctgttgggcactgacaattccgtggtgttgtcggggaagctgacgtcctttcca
tggctgctcgcctgtgttgccacctggattctgcgcgggacgtccttctgctacgtcccttcggccctcaatccagcggaccttccttcccgcgg
cctgctgccggctctgcggcctcttccgcgtcttcgccttcgccctcagacgagtcggatctccctttgggccgcctccccgcctggaattcgag
ctcggtacctttaagaccaatgacttacaaggcagctgtagatcttagccactttttaaaagaaaaggggggactggaagggctaattcact
cccaacgaagacaagatctgctttttgcttgtactgggtctctctggttagaccagatctgagcctgggagctctctggctaactagggaaccc
actgcttaagcctcaataaagcttgccttgagtgcttcaagtagtgtgtgcccgtctgttgtgtgactctggtaactagagatccctcagaccct
tttagtcagtgtggaaaatctctagcagtagtagttcatgtcatcttattattcagtatttataacttgcaaagaaatgaatatcagagagtgag
aggaacttgtttattgcagcttataatggttacaaataaagcaatagcatcacaaatttcacaaataaagcatttttttcactgcattctagttgt
ggtttgtccaaactcatcaatgtatcttatcatgtctggctctagctatcccgcccctaactccgcccagttccgcccattctccgccccatggct
gactaattttttttatttatgcagaggccgaggccgcctcggcctctgagctattccagaagtagtgaggaggcttttttggaggcctaggcttt
gcgtcgagacgtacccaattcgccctatagtgagtcgtattacgcgcgctcactggccgtcgttttacaacgtcgtgactgggaaaaccctgg
cgttacccaacttaatcgccttgcagcacatccccctttcgccagctggcgtaatagcgaagaggcccgcaccgatcgcccttcccaacagtt
gcgcagcctgaatggcgaatggcgcgacgcgccctgtagcggcgcattaagcgcggcgggtgtggtggttacgcgcagcgtgaccgctaca
cttgccagcgccctagcgcccgctcctttcgctttcttcccttcctttctcgccacgttcgccggctttccccgtcaagctctaaatcgggggctcc
ctttagggttccgatttagtgctttacggcacctcgaccccaaaaaacttgattagggtgatggttcacgtagtgggccatcgccctgatagac
ggtttttcgccctttgacgttggagtccacgttctttaatagtggactcttgttccaaactggaacaacactcaaccctatctcggtctattctttt
gatttataagggattttgccgatttcggcctattggttaaaaaatgagctgatttaacaaaaatttaacgcgaattttaacaaaatattaacgtt
tacaatttcc
