# Supplementary figures and images for: Visualization of aging-associated chromatin alterations with an engineered TALE system
Source: Cell Res. 2017 Jan 31;27(4):483–504. doi: 10.1038/cr.2017.18 (PMC5385610; doi:10.1038/cr.2017.18)

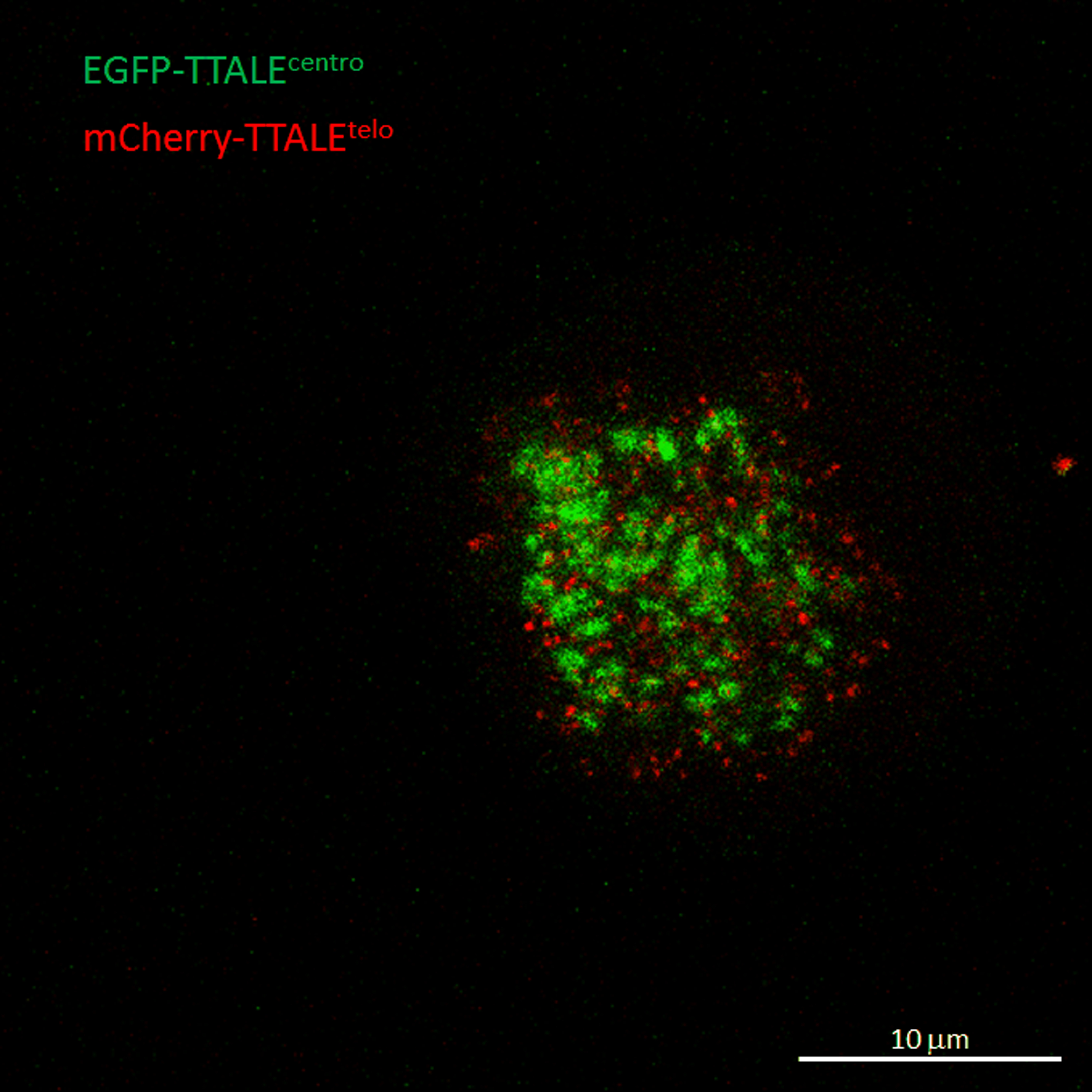

Supplement: Supplementary information, Movie S1 — TTALE-based live cell imaging of telomeres and centromeres in mitotic HeLa cells cotransfected with EGFP-TTALEcentro (green) and mCherry-TTALEtelo (red) [file cr201718x16.tif]
